# Twenty years of real-world data to estimate chronic kidney disease prevalence and staging in an unselected population

**DOI:** 10.1093/ckj/sfac206

**Published:** 2022-09-12

**Authors:** Carla Santos-Araújo, Luís Mendonça, Daniel Seabra Carvalho, Filipa Bernardo, Marisa Pardal, João Couceiro, Hugo Martinho, Cristina Gavina, Tiago Taveira-Gomes, Ricardo Jorge Dinis-Oliveira

**Affiliations:** UnIC@RISE, Department of Surgery and Physiology, Faculty of Medicine of the University of Porto, Porto, Portugal; Nephrology Department, Pedro Hispano Hospital, Senhora da Hora, Matosinhos, Portugal; UnIC@RISE, Department of Surgery and Physiology, Faculty of Medicine of the University of Porto, Porto, Portugal; Nephrology Department, Centro Hospitalar Universitário São João, EPE, Porto, Portugal; Department of Community Medicine, Information and Decision in Health, Faculty of Medicine, University of Porto, Porto, Portugal; Medical Department, AstraZeneca, Barcarena, Portugal; Medical Department, AstraZeneca, Barcarena, Portugal; Medical Department, AstraZeneca, Barcarena, Portugal; Medical Department, AstraZeneca, Barcarena, Portugal; Cardiology Department, Pedro Hispano Hospital, Senhora da Hora, Matosinhos, Portugal; Department of Community Medicine, Information and Decision in Health, Faculty of Medicine, University of Porto, Porto, Portugal; MTG Research and Development Lab, Porto, Portugal; Center for Health Technology and Services Research, Porto, Portugal; Faculty of Health Sciences, University Fernando Pessoa, Porto, Portugal; MTG Research and Development Lab, Porto, Portugal; Toxicology Research Unit, University Institute of Health Sciences, Advanced Polytechnic and University Cooperative, CRL, Gandra, Portugal; UCIBIO-REQUIMTE, Laboratory of Toxicology, Department of Biological Sciences, Faculty of Pharmacy, University of Porto, Porto, Portugal; Department of Public Health and Forensic Sciences, and Medical Education, Faculty of Medicine, University of Porto, Porto, Portugal

**Keywords:** albuminuria, chronic kidney disease, comorbidities, glomerular filtration rate, Portugal, prevalence

## Abstract

Chronic kidney disease (CKD) represents a global public health burden, but its true prevalence is not fully characterized in the majority of countries. We studied the CKD prevalence in adult users of the primary, secondary and tertiary healthcare units of an integrated health region in northern Portugal (*n* = 136 993; representing ∼90% of the region’s adult population). Of these, 45 983 (33.6%) had at least two estimated glomerular filtration rate (eGFR) assessments and 30 534 (22.2%) had at least two urinary albumin:creatinine ratio (UACR) assessments separated by at least 3 months. CKD was defined according to the Kidney Disease: Improving Global Outcomes (KDIGO) guidelines as a persistent decrease in eGFR (<60 ml/min/1.73 m^2^) and/or an increase in UACR (≥30 mg/g). The estimated overall prevalence of CKD was 9.8% and was higher in females (5.5%) than males (4.2%). From these, it was possible to stratify 4.7% according to KDIGO guidelines. The prevalence of CKD was higher in older patients (especially in patients >70 years old) and in patients with comorbidities.

This is the first real-world-based study to characterize CKD prevalence in a large, unselected Portuguese population. It probably provides the nearest estimate of the true CKD prevalence and may help healthcare providers to guide CKD-related policies and strategies focused on prevention and on the improvement of cardiovascular disease and other outcomes.

## Graphical Abstract

**Graphical Abstract:**
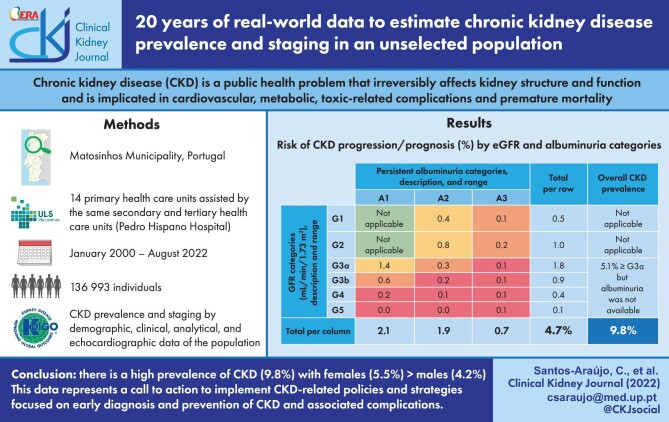

## INTRODUCTION

Chronic kidney disease (CKD) is a general term for heterogeneous disorders that irreversibly affect kidney structure and function for >3 months and is implicated in cardiovascular, metabolic, endocrine and xenobiotic toxicity-related complications and in premature mortality [1–3]. CKD is typically defined as a decreased glomerular filtration rate (GFR) and/or increased albuminuria. The worldwide prevalence of CKD was estimated to be ∼11–13% [4] and globally in 2017 it was estimated that nearly 700 million persons had CKD and 1.2 million people died from CKD-related disorders [5]. Moreover, the burden of CKD is expected to increase in the future, especially due to the increase in global aging and the increasing prevalence of hypertension, obesity and type 2 diabetes mellitus (T2DM) [6, 7].

Fortunately, the development of CKD comorbidities can be delayed or prevented if they are rapidly detected [8]. To achieve this, CKD epidemiology needs to be carefully assessed. However, data regarding CKD prevalence and staging in the early stages and morbidity and mortality are scarce or non-existent in many countries [5]. Moreover, even where data are available, a significant heterogeneity of CKD prevalence between regions exists, probably due to disparities in clinical risk factors, methodologies used for creatinine determination, formulas for calculation of estimated GFR (eGFR) and statistical approaches [4, 9, 10]. For instance, across the European population there were considerable differences in the prevalence of both CKD stages 1–5 and CKD stages 3–5 [11]. In the adult general population of the USA, the adjusted prevalence of CKD stages 3–5 ranged from 4.8% to 11.8% in the Northeast and Midwest, respectively [12]. In a very recent publication, the CaReMe CKD study was designed to estimate the prevalence of CKD, key clinical adverse outcomes and costs of CKD across 11 countries [13]. Relevant individual-level data for a cohort of 2.4 million CKD patients was obtained from digital healthcare systems and revealed a pooled prevalence of possible CKD of 10% and a confirmed prevalence of CKD ranging from 5.6 to 9.8% [13].

Specifically in Portugal, the PREVADIAB study showed a prevalence of CKD stages 3–5 of 6.1% [14, 15]. Although this study was a very relevant starting point, some limitations can be highlighted, such as the absence of data on the estimation of prevalence of CKD stages 1 and 2, inclusion of subjects only 20–79 years of age and non-compliance with the criterion for verifying kidney disease chronicity by 3 months after diagnosis, as recommended by the Kidney Disease: Improving Global Outcomes (KDIGO) guidelines [8]. To overcome the reported limitations, more recently the RENA study aimed to estimate the prevalence of CKD and characterize patients on a national level [16]. This cross-sectional study included users of primary health care units (PCHUs) ≥18 years of age and the sociodemographic and clinical data were recorded through a structured questionnaire. Results showed a higher CKD prevalence compared with the global and European previously reported average. Nevertheless, the applied methodology based on voluntary participation of PCHU users presenting in the waiting room, offers some constraints. Indeed, as highlighted by the authors, despite all efforts, this approach may have compromised the global picture by unbiasing results, as the PCHU attendees are not representative of the real population since attendees typically possess multiple comorbidities [17, 18].

Taking this into consideration, the current study aimed to fully characterize the prevalence of CKD in a non-selected population of a group of PCHUs supported by a unique secondary and tertiary care health unit (STCHU) in northern Portugal and simultaneously compare the variation in CKD prevalence and staging by demographic, clinical, analytical and echocardiographic data for the population.

## MATERIALS AND METHODS

### Study design

This is an observational cohort and cross-sectional study performed in the Health Local Unit of Matosinhos (Unidade Local de Saúde de Matosinhos; ULSM), a regional health system in the district of Matosinhos in northern Portugal, including 14 PCHUs assisted by the same STCHU, the Pedro Hispano Hospital. We selected all persons ≥18 years of age who were seen at least once in the healthcare units in the 3 years before the index date (31 May 2022). A 22-year period of data analyses (since 1 January 2000) was applied. A total of 136 993 users matching the inclusion criteria were enrolled, representing ∼90% of the adult population of the geographic region of Matosinhos, according to the 2021 Portuguese census (the eighth most inhabited municipality in the country and the fourth in the northern region). In other words, ∼90% of the adult population of Matosinhos was attended at a healthcare unit at least once in the 3 years before data access. Data access for analysis was granted after approval by the Ethical Committee and Data Protection Officer of the ULSM [approval 34/CE/JAS of 23 April 2020 (original) and 64/CE/JAS of 10 July 2020 (addenda)]. Following the Health Insurance Portability and Accountability Act Safe Harbor Standard, de-identified data regarding age, gender, body mass index (BMI), waist circumference, systolic blood pressure, diastolic blood pressure, echocardiography and laboratory measurements (including general, iron, diabetes, lipid, liver, heart, thyroid and kidney panels) and general, cardiovascular and bone comorbidities classified by the International Classification of Diseases, Ninth and Tenth Revisions (ICD-9 and ICD-10) codes and current cardiovascular, diabetes and bone disease medications registered according to the Anatomical Therapeutic Chemical Classification System were extracted from electronic health records. Plasmatic creatinine determination was performed in the same laboratory and by the same method for all samples and was used for eGFR calculation. Urinary albumin:creatinine ratio (UACR) was used for albuminuria detection, as defined by the KDIGO guidelines. Only those patients with two or more tests for serum creatinine and/or albuminuria, at least 3 months apart, were included in this study. Patients with only one CKD test were not included in the prevalence calculations.

Nutritional status was classified in the following categories according to BMI: underweight (<18.5 kg/m^2^), normal weight (18.5–24.9 kg/m^2^), pre-obesity (25.0–29.9 kg/m^2^) and obesity class 1 (30–34.9 kg/m^2^), class 2 (35–39.9 kg/m^2^) and class 3 (≥40 kg/m^2^).

### CKD definitions and calculations

CKD stages 1–5 were defined and classified based on the KDIGO guidelines [8] as either decreased eGFR (<60 ml/min/1.73 m^2^) or the presence of albuminuria assessed as UACR ≥30 mg/g or 3 mg/mmol for >3 months. eGFR was estimated by the Chronic Kidney Disease Epidemiology Collaboration (CKD-EPI) equation, considering gender and serum creatinine [19]: eGFR (ml/min/1.73 m^2^) = 141 × min(SCr/κ, 1)^α^ × max(SCr/κ, 1)^−1.209^ × 0.993^Age^ × [1.018 (if female)] or [1.159 (if Black)], where SCr is serum creatinine (mg/dl), α is 0.7 for females and 0.9 for males, κ is −0.329 for females and −0.411 for males, min indicates the minimum of SCr/κ or 1 and max indicates the maximum of SCr/κ or 1. As an additional analysis, the creatinine clearance was estimated by the Cockcroft–Gault (CG) equation as previously described [10]. eGFR categories were defined as follows: G_1_, normal or high (≥90 ml/min/1.73 m^2^); G_2_, mildly decreased (60–89 ml/min/1.73 m^2^); G_3a_, mildly to moderately decreased (45–59 ml/min/1.73 m^2^); G_3b_, moderately to severely decreased (30–44 ml/min/1.73 m^2^); G_4_, severely decreased (15–29 ml/min/1.73 m^2^); and G_5_, kidney failure (<15 ml/min/1.73 m^2^). Albuminuria was defined in three categories: A_1_, normal to mildly increased (UACR <30 mg/g); A_2_, moderately increased (UACR 30–300 mg/g); and A_3_, severely increased (UACR ≥300 mg/g). Patients in stages G1/A1 and G2/A1 were not characterized for CKD since other data regarding renal lesions, such as echography, urinary sediment and renal biopsy reports, were not available.

### Statistical analysis

Statistical analyses were performed using Spark 3.1.0 (Apache Software Foundation, Wilmington, DE, USA). Normally distributed variables were presented using mean and respective percentages and non-normally distributed data as medians with interquartile ranges (IQRs). Overall population and CKD staging were stratified into the 50–60, 60–70, 70–80 and >80-year age groups. The prevalence of CKD was estimated as the number with confirmed CKD divided by the number of all individuals registered in the health units enrolled at the time of data access.

## RESULTS

### Characterization of population

A population of 136 993 individuals, 59 867 (43.7%) males and 77 126 (56.3%) females, were enrolled in this study. A median age of 52.0 years (IQR 30.0) was recorded. Of note, hypertension and T2DM had a prevalence of 42.5% (*n* = 58 200) and 23.0% (*n* = 31 494), respectively, while obesity was present in 20.3% of the population studied (*n* = 27 835).

### Prevalence and characterization of the CKD population

**Figure 1:**
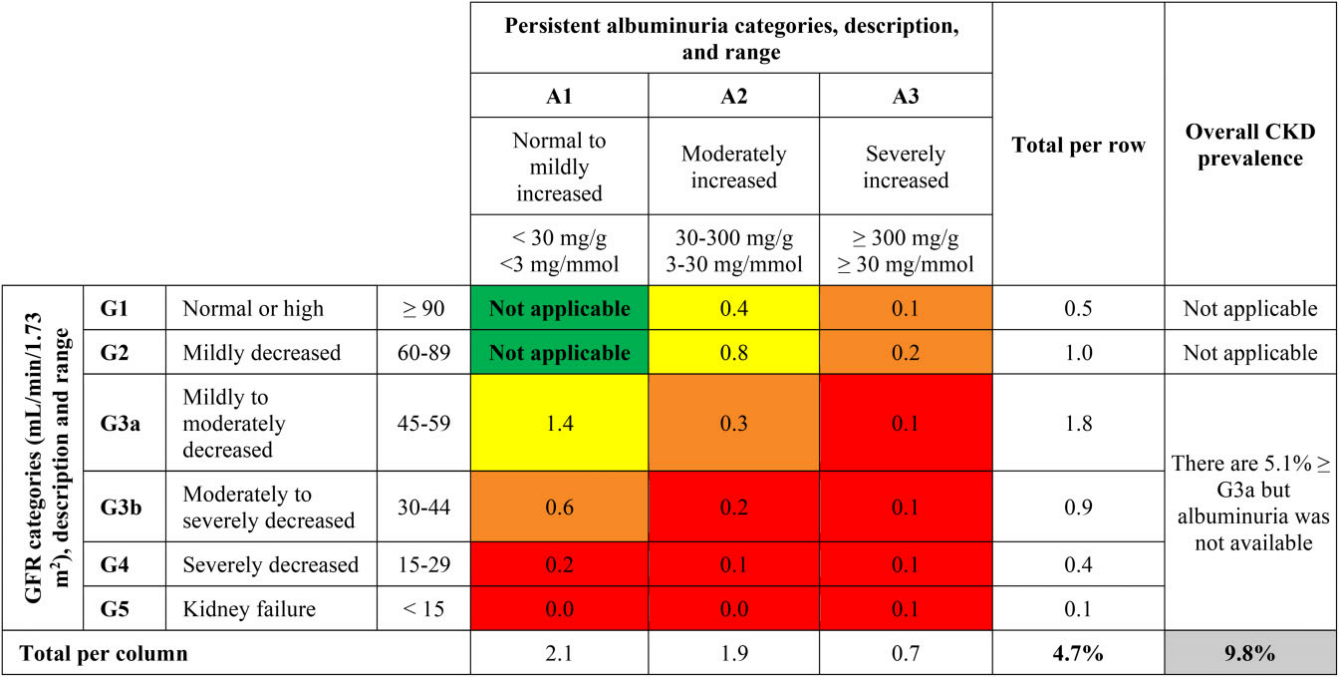
Risk of CKD progression/prognosis (%) by eGFR and albuminuria categories. Overall CKD prevalence is presented for all patients with two eGFR values <60 ml/min/1.73 m^2^ (G3–G5) and/or two UACR values ≥30 mg/g (A2–A3) persistent for at least 3 months. From these, 4.7% of patients were possible to be stratified according to KDIGO guidelines and the CKD risk was defined as follow: green, low risk/no CKD in absence of markers of kidney disease; yellow, moderately increased risk; orange, high risk; red, very high risk. According to the KDIGO, patients in stage G1/A1 and G2/A1 were not characterized for CKD since other data for renal lesions, such as echography, urinary sediment and renal biopsy reports, were not available. Data are presented for percentages of the overall population.

To reduce the possibility of CKD false-positive results, we evaluated and confirmed CKD by assessing eGFR and UACR at least twice at least 3 months apart. In total, 45 983 (33.6%) persons had at least two eGFR assessments (Table 1, Supplementary Tables S1 and S2) and 30 534 (22.3%) had at least two UACR assessments separated by at least 3 months (Table 2, Supplementary Tables S3 and S4). Tables 1 and 2 present a detailed characterization of CKD in the population according to the KDIGO guidelines, using CKD-EPI and UACR, respectively. Individual characterizations for female and male populations are provided in Supplementary Figures S1 and S2, respectively. According to the KDIGO guidelines, which define CKD as two eGFR values <60 ml/min/1.73 m^2^ (G3–G5) and/or two UACR values ≥30 mg/g (A2–A3) persistent for at least 3 months, the estimated overall prevalence of CKD was 9.8% and was higher in females (5.5%) than males (4.2%). From these, 4.7% could be stratified according to the KDIGO guidelines (Figure 1). The prevalence of CKD was higher in older patients (especially in patients >70 years old) and in patients with comorbidities. We were also able to identify a significant percentage of patients [27.2% (*n* = 37 292)] with an eGFR of 60–89 ml/min/1.73 m^2^. The prevalence of CKD, using two measurements of creatinine clearance calculated by the CG equation was 11.3% (detailed data not shown).

**Table 1:** Detailed characterization of the CKD population according to the KDIGO guidelines using the CKD-EPI equation.

	eGFR ≥90 ml/min/1.73 m^2^ [*n* = 652 (0.4%)]		eGFR 60–89 ml/min/1.73 m^2^ [*n* = 37 292 (27.2%)]		eGFR 45–59 ml/min/1.73 m^2^ [*n* = 4322 (3.1%)]		eGFR 30–44 ml/min/1.73 m^2^ [*n* = 2276 (1.7%)]		eGFR 15–29 ml/min/1.73 m^2^ [*n* = 1038 (0.7%)]		eGFR <15 ml/min/1.73 m^2^ [*n* = 403 (0.3%)]	
Characteristics	*n*	%	*n*	%	*n*	%	*n*	%	*n*	%	*n*	%
Sociodemographic characteristics												
Male	417	64.0	15 220	40.8	1812	41.9	873	38.3	403	38.8	161	40.0
Female	235	36.0	22 072	59.2	2510	58.1	1403	61.6	635	61.2	242	60.1
Age (years)	57.5 (P50)	12.0 (IQR)	68.0 (P50)	20.0 (IQR)	78.0 (P50)	14.0 (IQR)	81.0 (P50)	14.0 (IQR)	83.0 (P50)	15.0 (IQR)	80.0 (P50)	18.0 (IQR)
20–79	59 856	99.7	30 009	84.0	2247	54.8	903	43.8	347	42.3	135	62.5
50–60	242	37.1	6148	16.5	166	3.8	57	2.5	30	2.9	26	6.5
60–70	222	34.0	8708	23.4	672	15.5	250	11.0	105	10.1	63	15.6
70–80	37	5.7	10 967	29.4	1403	32.5	638	28.0	233	22.4	95	23.6
>80	7	1.1	6089	16.3	2013	46.6	1314	57.7	644	62.0	206	51.1
BMI (kg/m^2^)	*n*	%	*n*	%	*n*	%	*n*	%	*n*	%	*n*	%
<18.5	5	0.8	454	1.2	56	1.3	40	1.8	21	2.0	10	2.5
18.5–25.0	104	16.0	10 757	28.8	1092	25.3	540	23.7	260	25.0	114	28.3
25.0–30.0	241	37.0	15 083	40.4	1734	40.1	884	38.8	360	34.7	147	36.5
30.0–35.0	178	27.3	6927	18.6	913	21.1	482	21.2	222	21.4	65	16.1
≥20	110	16.9	2375	6.4	350	8.1	221	9.7	102	9.8	28	6.9
Clinical measurements	P50	IQR	P50	IQR	P50	IQR	P50	IQR	P50	IQR	P50	IQR
Weight (kg)	81.0	23.0	72.0	18.0	72.0	18.0	72.0	19.0	71.0	19.0	69.5	20.0
BMI (kg/m^2^)	29.4	6.8	27.0	5.9	27.4	6.1	27.8	6.4	27.6	7.1	27.0	6.4
Waist circumference (cm)	103.0	16.0	98.0	15.0	100.0	15.0	102.0	17.0	103.0	17.0	103.0	15.0
SBP (mmHg)	138.0	19.0	134.0	18.0	137.0	19.0	137.0	20.0	137.0	25.0	139.0	26.0
DBP (mmHg)	83.0	11.0	79.0	13.0	76.0	13.0	75.0	15.0	74.0	15.0	73.0	19.0
Echocardiography measurements	P50	IQR	P50	IQR	P50	IQR	P50	IQR	P50	IQR	P50	IQR
Left atrial volume (ml)	40.0	6.0	39.0	7.0	40.0	7.0	41.0	7.0	42.0	8.0	42.0	8.5
Left atrial volume index (ml/m^2^)	20.9	3.5	21.9	4.2	22.7	4.6	23.4	4.9	23.9	5.4	24.0	5.4
Left ventricular mass (g)	139.6	43.9	134.2	40.9	145.0	42.9	146.4	44.6	148.3	50.4	157.3	50.4
Left ventricular mass index (g/m^2^)	73.2	18.1	75.5	21.6	80.9	23.9	82.2	25.1	85.6	27.3	88.1	29.2
Left atrial diameter (mm)	50.0	6.5	49.0	6.0	49.0	7.0	50.0	7.0	50.0	7.5	51.0	8.0
Ejection fraction (%)	61.0	7.5	62.0	8.0	61.0	9.0	60.0	9.0	60.0	12.0	58.0	13.0
Left ventricular posterior wall thickness (ml)	9.0	1.0	9.0	2.0	9.0	1.0	9.0	1.0	10.0	2.0	10.0	2.0
Interventricular septum thickness (mm)	10.0	2.0	10.0	2.0	11.0	2.0	11.0	2.0	11.0	2.0	11.0	3.0
Laboratory measurements	P50	IQR	P50	IQR	P50	IQR	P50	IQR	P50	IQR	P50	IQR
Haemoglobin (g/dl)	15.1	16.3	14.6	16.5	13.9	16.8	12.9	3.8	11.7	2.9	10.7	2.6
Sodium (mEq/l)	140.0	3.0	140.0	2.2	140.0	4.0	140.0	4.0	140.0	4.0	139.0	6.0
Potassium (mEq/l	4.3	0.5	4.3	0.5	4.4	0.6	4.5	0.7	4.5	0.8	4.7	1.2
Phosphate (mg/dl)	3.3	0.9	3.3	0.7	3.3	0.8	3.4	0.8	3.6	1.0	4.3	1.8
Magnesium (mg/dl)	2.0	0.3	2.1	0.3	2.0	0.4	2.0	0.4	2.1	0.4	2.1	0.4
Calcium (mg/dl)	9.4	0.6	9.4	0.6	9.4	0.7	9.4	0.8	9.3	0.8	8.9	1.0
Vitamin D (ng/ml)	17.0	11.0	20.0	14.5	17.0	15.9	17.0	16.0	19.0	21.0	15.0	16.0
Uric acid (µg/dl)	5.4	2.2	5.1	1.9	6.0	2.3	6.5	2.5	7.2	3.1	7.3	3.0
Creatine kinase (IU/l)	86.0	85.5	85.0	69.0	79.0	71.0	75.5	70.5	75.0	78.3	80.0	85.5
Iron (µg/l)	72.0	52.8	81.0	45.0	70.0	40.0	65.0	40.0	59.0	38.0	50.0	39.8
Transferrin (µg/l)	248.0	100.0	249.0	75.0	238.0	82.0	237.0	77.5	225.5	87.3	197.5	81.8
Total iron binding capacity (µg/l)	313.5	100.8	302.0	83.0	289.0	85.0	285.0	83.0	263.0	91.0	241.0	81.0
Parathyroid hormone (pg/ml)	38.8	21.1	50.4	34.4	71.1	52.6	90.3	72.2	127.5	111.6	197.1	209.6
Glucose (mg/dl)	123.0	62.8	99.0	29.0	108.3	43.0	116.0	52.5	119.8	65.0	119.0	65.5
HbA1c (%)	7.8	34.9	6.2	28.4	6.6	30.2	7.2	32.1	7.4	32.2	7.3	30.3
LDL cholesterol (mg/dl)	113.0	48.0	118.2	45.0	108.0	44.0	102.2	44.0	98.0	45.9	100.0	50.0
HDL cholesterol (mg/dl)	43.0	16.0	48.0	16.0	44.0	16.0	42.0	16.0	39.0	17.0	40.0	16.6
Non-HDL cholesterol (mg/dl)	139.0	57.0	139.0	48.0	131.0	46.0	128.0	49.0	122.0	48.0	126.0	52.1
Total cholesterol (mg/dl)	186.0	57.0	190.0	51.0	178.0	51.0	173.0	52.0	166.0	54.3	167.0	55.0
Triglycerides (mg/dl)	127.5	83.0	103.0	61.0	117.0	66.0	123.0	72.0	122.0	73.0	122.0	74.5
Brain natriuretic peptide (pg/ml)	47.8	114.0	91.2	160.8	153.5	305.8	160.7	325.4	244.3	471.1	640.5	1132.7
NT-pro-brain natriuretic peptide (pg/ml)	303.1	0.0	721.4	2053.1	1679.5	3412.8	2968.2	10 519.2	4509.3	6148.2	16 884.9	31 300.0
Albumin (g/dl)	4.4	0.8	4.5	42.5	4.3	1.1	4.2	0.9	3.9	0.9	3.5	1.0
Bilirubin (mg/dl)	0.6	0.4	0.6	0.3	0.6	0.4	0.6	0.4	0.5	0.4	0.5	0.4
ALT (IU/l)	25.0	18.0	19.0	12.0	17.0	11.0	16.0	11.0	16.0	11.0	17.0	18.0
AST (IU/l)	21.0	10.0	20.0	7.0	20.0	8.0	20.0	10.0	20.0	11.0	21.0	16.0
Alkaline phosphatase (IU/l)	73.0	32.0	70.0	30.0	75.0	35.0	79.0	38.0	90.0	52.0	93.0	56.0
TSH (IU/ml)	1.5	1.1	1.7	1.2	1.7	1.4	1.7	1.4	1.8	1.7	1.7	1.5
T3 (µg/dl)	2.9	0.7	2.8	0.7	2.7	0.7	2.6	0.7	2.5	0.8	2.4	0.8
T4 (µg/dl)	1.0	0.2	1.0	0.2	1.0	0.2	1.0	0.2	1.0	0.2	1.0	0.3
Creatinine (mg/dl)	0.7	0.1	0.8	0.2	1.1	0.3	1.4	0.4	2.1	0.7	4.1	1.0
Comorbidities	P50	IQR	P50	IQR	P50	IQR	P50	IQR	P50	IQR	P50	IQR
Obesity	288	44.2	9302	24.9	1263	29.2	703	30.9	324	31.2	93	23.1
Hypercholesterolemia	292	44.8	18 118	48.6	1553	35.9	704	30.9	269	25.9	119	29.5
T2DM	459	70.4	12 987	34.8	2303	53.3	1482	65.1	761	73.3	306	75.9
Structural heart disease	142	21.8	6451	17.3	1438	33.3	1047	46.0	646	62.2	298	73.9
Microvascular disease	80	12.3	1924	5.2	434	10.0	406	17.8	276	26.6	158	39.2
Cardiovascular disease	608	93.3	27 155	72.8	4322	100.0	2276	100.0	1 038	100.0	403	100.0
Hypertension	595	91.3	25 431	68.2	3881	89.8	2100	92.3	938	90.4	356	88.3
Atrial fibrillation	25	3.8	2356	6.3	739	17.1	503	22.1	345	33.2	123	30.5
Stable angina	45	6.9	1587	4.3	396	9.2	295	13.0	176	17.0	97	24.1
Transient ischaemic attack	11	1.7	406	1.1	89	2.1	63	2.8	38	3.7	12	3.0
Atherosclerotic disease	126	19.3	4858	13.0	1 142	26.4	753	33.1	452	43.5	204	50.6
Unstable angina	20	3.1	808	2.2	213	4.9	140	6.2	78	7.5	34	8.4
Myocardial infarction	40	6.1	1140	3.1	314	7.3	220	9.7	141	13.6	93	23.1
Stroke	68	10.4	3084	8.3	688	15.9	447	19.6	276	26.6	115	28.5
Ischaemic stroke	60	9.2	2472	6.6	563	13.0	382	16.8	246	23.7	100	24.8
Haemorrhagic stroke	3	0.5	278	0.7	56	1.3	33	1.4	27	2.6	14	3.5
Peripheral artery disease	42	6.4	1437	3.9	414	9.6	259	11.4	156	15.0	84	20.8
Heart failure	10	1.5	899	2.4	383	8.9	345	15.2	278	26.8	163	40.4
Preserved	8	1.2	748	2.0	314	7.3	273	12.0	223	21.5	124	30.8
Midrange	2	0.3	146	0.4	64	1.5	63	2.8	44	4.2	29	7.2
Reduced	0	0.0	5	0.0	5	0.1	9	0.4	11	1.1	10	2.5
Cardiovascular medications	*n*	%	*n*	%	*n*	%	*n*	%	*n*	%	*n*	%
Renin–angiotensin system agents	571	87.6	20 883	56.0	3700	85.6	2057	90.4	934	90.0	360	89.3
Angiotensin-converting enzyme inhibitors	435	66.7	14 481	38.8	2702	62.5	1539	67.6	680	65.5	270	67.0
Angiotensin receptor blockers	336	51.5	13 002	34.9	2461	56.9	1434	63.0	684	65.9	267	66.3
Angiotensin receptor–neprilysin inhibitors	1	0.2	48	0.1	29	0.7	19	0.8	8	0.8	0	0.0
Diuretics	219	33.6	11 181	30.0	2345	54.3	1602	70.4	844	81.3	344	85.4
Thiazides	6	0.9	194	0.5	37	0.9	20	0.9	6	0.6	1	0.2
Sulfonamides	102	15.6	5534	14.8	1519	35.1	1256	55.2	764	73.6	331	82.1
Aldosterone antagonists	22	3.4	1223	3.3	320	7.4	278	12.2	166	16.0	50	12.4
Antiplatelets	227	34.8	9687	26.0	2073	48.0	1263	55.5	615	59.2	252	62.5
Low-dose aspirin	216	33.1	8869	23.8	1893	43.8	1156	50.8	562	54.1	237	58.8
P2Y_12_ antagonists	70	10.7	2677	7.2	620	14.3	418	18.4	209	20.1	89	22.1
Anticoagulants	46	7.1	2974	8.0	806	18.6	530	23.3	294	28.3	107	26.6
Vitamin K antagonists	32	4.9	1534	4.1	471	10.9	335	14.7	212	20.4	92	22.8
Novel oral anticoagulants	22	3.4	1801	4.8	460	10.6	280	12.3	138	13.3	20	5.0
Calcium channel blockers	223	34.2	7518	20.2	1823	42.2	1265	55.6	648	62.4	287	71.2
Beta blockers	228	35.0	9524	25.5	1856	42.9	1178	51.8	605	58.3	234	58.1
Nitrates	49	7.5	1911	5.1	493	11.4	395	17.4	235	22.6	111	27.5
Diabetes medications	*n*	%	*n*	%	*n*	%	*n*	%	*n*	%	*n*	%
Glucose-lowering drugs	402	61.7	8880	23.8	1669	38.6	1 058	46.5	537	51.7	211	52.4
Excluding insulins	389	59.7	8778	23.5	1653	38.2	1 037	45.6	521	50.2	200	49.6
Biguanides	377	57.8	8279	22.2	1528	35.4	949	41.7	452	43.5	177	43.9
Sodium–glucose cotransporter-2 inhibitors	75	11.5	923	2.5	178	4.1	72	3.2	22	2.1	2	0.5
Glucagon-like peptide-1 receptor agonists	40	6.1	304	0.8	65	1.5	59	2.6	24	2.3	2	0.5
Dipeptidyl peptidase-4 inhibitors	219	33.6	3627	9.7	939	21.7	710	31.2	392	37.8	146	36.2
Glitazones	40	6.1	696	1.9	196	4.5	159	7.0	93	9.0	34	8.4
Sulfonylureas	172	26.4	3209	8.6	836	19.3	591	26.0	296	28.5	130	32.3
Metiglinides	4	0.6	97	0.3	23	0.5	23	1.0	12	1.2	6	1.5
Glucosidase inhibitors	34	5.2	589	1.6	173	4.0	137	6.0	60	5.8	38	9.4
Insulins	111	17.0	1184	3.2	362	8.4	383	16.8	260	25.0	143	35.5
Long acting	81	12.4	807	2.2	245	5.7	286	12.6	186	17.9	92	22.8
Intermediate acting	49	7.5	520	1.4	157	3.6	161	7.1	127	12.2	79	19.6
Fast acting	33	5.1	342	0.9	87	2.0	98	4.3	71	6.8	46	11.4
Premixed	32	4.9	339	0.9	109	2.5	102	4.5	61	5.9	39	9.7
Bone medications	*n*	%	*n*	%	*n*	%	*n*	%	*n*	%	*n*	%
Bone disease medications	26	4.0	6365	17.1	967	22.4	483	21.2	189	18.2	45	11.2
Vitamin D	52	8.0	4331	11.6	677	15.7	525	23.1	390	37.6	135	33.5
Calcium	44	6.7	6427	17.2	925	21.4	464	20.4	219	21.1	85	21.1
Magnesium	80	12.3	5691	15.3	819	18.9	466	20.5	210	20.2	81	20.1
Oestrogens and progesterones	71	10.9	8161	21.9	777	18.0	379	16.7	149	14.4	38	9.4
Calcitonins	0	0.0	296	0.8	53	1.2	23	1.0	16	1.5	1	0.2

In total, 45 983 (33.5%) patients had at least two eGFR assessments. Percentages are presented for the overall population (*n* = 136 993).

ALT, alanine aminotransferase; AST, aspartate aminotransferase; DBP, diastolic blood pressure; HbA1c, haemoglobin A1c; HDL, high-density lipoprotein; LDL, low-density lipoprotein; P2Y12, chemoreceptor for adenosine diphosphate; P50, median; T3, triiodothyronine; T4, thyroxine; TSH, thyroid-stimulating hormone.

**Table 2:** Detailed characterization of the CKD population according to KDIGO guidelines using UACR (mg/g).

Characteristics	UACR <30 [*n* = 26 498 (19.3%)]		UACR 30–300 [*n* = 2932 (2.1%)]		UACR ≥300 [*n* = 1104 (0.8%)]	
	*n*	%	*n*	%	*n*	%
Sociodemographic characteristics						
Male	11 961	45.1	1529	52.2	665	60.2
Female	14 537	54.9	1403	47.9	439	39.8
Age (years)	68.0 (P50)	17.0 (IRQ)	73.0 (P50)	19.0 (IQR)	72.0 (P50)	18.0 (IQR)
20–79	22 152	83.6	1991	67.9	784	71.0
50–60	4729	17.8	376	12.8	128	11.6
60–70	7914	29.9	655	22.3	263	23.8
70–80	7293	27.5	795	27.1	323	29.3
>80	4322	16.3	939	32.0	319	28.9
BMI (kg/m^2^)	*n*	%	*n*	%	*n*	%
<18.5	211	0.8	39	1.3	16	1.4
18.5–25.0	5739	21.7	630	21.5	245	22.2
25.0–30.0	11 277	42.6	1191	40.6	436	39.5
30.0–35.0	6452	24.3	703	24.0	238	21.6
≥20	2576	9.7	325	11.1	141	12.8
Clinical measurements	P50	IQR	P50	IQR	P50	IQR
Weight (kg)	74.0	19.0	75.0	20.0	76.0	20.0
BMI (kg/m^2^)	28.1	5.9	28.3	6.4	28.3	6.7
Waist circumference (cm)	100.0	14.0	102.0	15.0	104.0	16.0
SBP (mmHg)	136.0	17.0	138.0	20.0	141.0	20.0
DBP (mmHg)	80.0	13.0	78.0	15.0	79.0	15.0
Echocardiography measurements	P50	IQR	P50	IQR	P50	IQR
Left atrial volume (ml)	39.0	7.0	41.0	7.0	41.0	7.0
Left atrial volume index (ml/m^2^)	21.8	4.1	23.2	5.1	22.6	4.5
Left ventricular mass (g)	136.9	39.8	145.5	43.2	154.2	50.8
Left ventricular mass index (g/m^2^)	75.7	21.1	81.7	24.2	85.5	27.5
Left atrial diameter (mm)	49.0	7.0	50.0	6.0	50.0	7.0
Ejection fraction (%)	62.0	8.0	60.0	8.0	60.0	9.8
Left ventricular posterior wall thickness (ml)	9.0	2.0	9.0	1.0	10.0	2.0
Interventricular septum thickness (mm)	10.0	2.0	11.0	2.0	11.0	3.0
Laboratory measurements	P50	IQR	P50	IQR	P50	IQR
Haemoglobin (g/dl)	14.7	16.7	14.0	13.1	13.1	4.2
Sodium (mEq/l)	140.0	3.0	140.0	4.0	140.0	4.0
Potassium (mEq/l	4.3	0.6	4.4	0.6	4.5	0.8
Phosphate (mg/dl)	3.3	0.7	3.4	0.9	3.6	1.0
Magnesium (mg/dl)	2.1	0.3	2.0	0.4	2.0	0.4
Calcium (mg/dl)	9.4	0.6	9.4	0.8	9.3	0.9
Vitamin D (ng/ml)	19.0	15.0	17.0	16.0	18.0	16.0
Uric acid (µg/dl)	5.3	2.0	5.9	2.4	6.5	2.6
Creatine kinase (IU/l)	85.0	70.0	79.0	73.0	79.0	83.0
Iron (µg/l)	79.0	45.0	65.0	43.0	63.0	44.0
Transferrin (µg/l)	252.0	78.0	242.0	85.0	224.0	80.0
Total iron binding capacity (µg/l)	301.0	82.0	287.0	94.0	266.0	81.0
Parathyroid hormone (pg/ml)	58.3	49.9	84.2	105.1	123.4	132.3
Glucose (mg/dl)	106.0	38.2	127.0	61.8	131.2	72.5
HbA1c (%)	6.5	30.3	7.5	33.9	8.1	33.9
LDL cholesterol (mg/dl)	114.0	43.0	102.0	45.0	99.5	50.3
HDL cholesterol (mg/dl)	47.0	16.0	43.0	16.0	41.0	18.0
Non-HDL cholesterol (mg/dl)	135.5	46.0	127.0	50.8	128.0	54.3
Total cholesterol (mg/dl)	184.0	50.0	172.0	54.0	172.0	58.0
Triglycerides (mg/dl)	108.0	64.0	121.0	75.0	132.0	91.0
Brain natriuretic peptide (pg/ml)	91.8	169.3	164.2	343.7	180.7	394.0
NT-pro-brain natriuretic peptide (pg/ml)	992.4	3069.6	1163.0	4274.0	4148.2	6934.5
Albumin (g/dl)	4.4	38.9	4.2	0.9	4.1	0.9
Bilirubin (mg/dl)	0.6	0.3	0.6	0.4	0.5	0.4
ALT (IU/l)	20.0	13.0	19.0	13.0	19.0	14.0
AST (IU/l)	20.0	8.0	20.0	9.0	20.0	10.0
Alkaline phosphatase (IU/l)	71.0	30.0	77.0	35.0	83.5	43.0
TSH (IU/ml)	1.6	1.2	1.6	1.2	1.7	1.4
T3 (µg/dl)	2.9	0.7	2.7	0.8	2.6	0.9
T4 (µg/dl)	1.0	0.2	1.0	0.2	1.0	0.2
Creatinine (mg/dl)	0.8	0.3	0.9	0.5	1.3	1.3
Comorbidities	*n*	%	*n*	%	*n*	%
Obesity	9028	34.1	1028	35.1	379	34.3
Hypercholesterolemia	11 501	43.4	935	31.9	387	35.1
T2DM	13 249	50.0	2214	75.5	933	84.5
Structural heart disease	5915	22.3	1186	40.5	615	55.7
Microvascular disease	1803	6.8	562	19.2	376	34.1
Cardiovascular disease	25 262	95.3	2872	98.0	1090	98.7
Hypertension	24 872	93.9	2800	95.5	1056	95.7
Atrial fibrillation	1898	7.2	505	17.2	202	18.3
CKD	3451	13.0	1093	37.3	682	61.8
Stable angina	1577	6.0	374	12.8	186	16.8
Transient ischaemic attack	352	1.3	82	2.8	36	3.3
Arterosclerotic disease	4268	16.1	923	31.5	432	39.1
Unstable angina	795	3.0	179	6.1	75	6.8
Myocardial infarction	1165	4.4	264	9.0	134	12.1
Stroke	2473	9.3	544	18.6	269	24.4
Ischaemic stroke	1981	7.5	462	15.8	228	20.7
Haemorrhagic stroke	212	0.8	56	1.9	22	2.0
Peripheral artery disease	1349	5.1	332	11.3	185	16.8
Heart failure	900	3.4	328	11.2	226	20.5
Preserved	744	2.8	262	8.9	190	17.2
Midrange	139	0.5	62	2.1	32	2.9
Reduced	17	0.1	4	0.1	4	0.4
Cardiovascular medications	*n*	%	*n*	%	*n*	%
Renin–angiotensin system agents	22 765	85.9	2706	92.3	1049	95.0
Angiotensin-converting enzyme inhibitors	15 981	60.3	2044	69.7	827	74.9
Angiotensin receptor blockers	14 315	54.0	1811	61.8	772	69.9
Angiotensin receptor–neprilysin inhibitors	53	0.2	12	0.4	5	0.5
Diuretics	10 863	41.0	1588	54.2	754	68.3
Thiazides	214	0.8	21	0.7	13	1.2
Sulfonamides	5026	19.0	1082	36.9	591	53.5
Aldosterone antagonists	1091	4.1	216	7.4	122	11.1
Antiplatelets	8669	32.7	1518	51.8	673	61.0
Low-dose aspirin	8002	30.2	1418	48.4	629	57.0
P2Y_12_ antagonists	2449	9.2	480	16.4	240	21.7
Anticoagulants	2385	9.0	549	18.7	223	20.2
Vitamin K antagonists	1236	4.7	379	12.9	158	14.3
Novel oral anticoagulants	1451	5.5	273	9.3	99	9.0
Calcium channel blockers	8126	30.7	1534	52.3	757	68.6
Beta blockers	8981	33.9	1338	45.6	590	53.4
Nitrates	1829	6.9	402	13.7	193	17.5
Diabetes medications	*n*	%	*n*	%	*n*	%
Glucose-lowering drugs	9706	36.6	1846	63.0	789	71.5
Excluding insulins	9565	36.1	1814	61.9	766	69.4
Biguanides	9166	34.6	1747	59.6	710	64.3
Sodium–glucose cotransporter-2 inhibitors	1096	4.1	291	9.9	76	6.9
Glucagon-like peptide-1 receptor agonists	397	1.5	101	3.4	63	5.7
Dipeptidyl peptidase-4 inhibitors	4187	15.8	1137	38.8	554	50.2
Glitazones	753	2.8	235	8.0	149	13.5
Sulfonylureas	3537	13.3	969	33.0	464	42.0
Metiglinides	105	0.4	34	1.2	18	1.6
Glucosidase inhibitors	571	2.2	222	7.6	110	10.0
Insulins	1310	4.9	596	20.3	383	34.7
Long acting	959	3.6	433	14.8	288	26.1
Intermediate acting	546	2.1	263	9.0	169	15.3
Fast acting	404	1.5	149	5.1	121	11.0
Premixed	366	1.4	146	5.0	106	9.6
Bone medications	*n*	%	*n*	%	*n*	%
Bone disease medications	4393	16.6	421	14.4	114	10.3
Vitamin D	3309	12.5	499	17.0	321	29.1
Calcium	4569	17.2	485	16.5	164	14.9
Magnesium	4382	16.5	486	16.6	198	17.9
Oestrogens and progesterones	6013	22.7	459	15.7	140	12.7
Calcitonins	216	0.8	24	0.8	5	0.5

In total, 30 534 (22.3%) patients had at least two UARC assessments. Percentages are presented for the overall population (*n* = 136 993).

ALT, alanine aminotransferase; AST, aspartate aminotransferase; DBP, diastolic blood pressure; HbA1c, haemoglobin A1c; HDL, high-density lipoprotein; LDL, low-density lipoprotein; P2Y12, chemoreceptor for adenosine diphosphate; P50, median; T3, triiodothyronine; T4, thyroxine; TSH, thyroid-stimulating hormone.

A significant increase in the prevalence of CKD was seen in the older age groups (Table 3). Of note, we also observed an increase in the prevalence of comorbidities with CKD stage, namely T2DM, structural heart disease, microvascular disease, familial hypercholesterolemia, cardiovascular disease, hypertension, atrial fibrillation, stable and unstable angina, atherosclerotic disease, myocardial infarction, ischaemic and haemorrhagic stroke, peripheral artery disease and heart failure. Renin–angiotensin system agents, diuretics, antiplatelet agents, calcium channel blockers, beta blockers, glucose-lowering drugs (excluding insulins) and magnesium were among the most prescribed drugs in CKD patients, a finding observed throughout all CKD stages.

**Table 3:** Risk of CKD progression/prognosis (%) by eGFR and albuminuria categories according to age.

	G1		G2		G3a			G3b			G4			G5				
Age (years)	A2	A3	A2	A3	A1	A2	A3	A1	A2	A3	A1	A2	A3	A1	A2	A3	Total per row	Overall CKD prevalence, %
18–44 (*n* = 50 708)	0.128	0.041	0.018	0.014	0.004	0.002	0.004	0.004	0.002	0.004	0	0.002	0.002	0	0	0	0.227	0.8
45–64 (*n* = 46 597)	0807	0.174	0.483	0.105	0.326	0.105	0.034	0.069	0.052	0.054	0.015	0.011	0.079	0	0.013	0.060	2.386	5.3
65–74 (*n* = 20 661)	0.474	0.044	1.781	0.455	2.623	0.586	0.198	0.711	0.295	0.276	0.160	0.150	0.179	0.010	0.019	0.213	8.175	15.7
≥75 (*n* = 19 027)	0.000	0.074	24.891	2.623	14.190	6.165	1.230	8.940	3.159	1.177	4.073	0.993	0.825	1.361	0.142	0.247	70.090	38.7

Overall CKD prevalence is presented for all patients with two eGFR values <60 ml/min/1.73 m^2^ (G3–G5) and/or two UACR values ≥30 mg/g (A2–A3) persistent for at least 3 months. According to the KDIGO guidelines, patients in stage G1/A1 and G2/A1 were not characterized for CKD since other data for renal lesions, such as echography, urinary sediment and renal biopsy reports, were not available. Percentage data were calculated considering the number of individuals of each class.

## DISCUSSION

CKD is a major worldwide public health problem and a relevant cost burden to healthcare systems. It is currently defined by abnormalities of kidney structure or function assessed by the eGFR, thresholds of albuminuria and duration of injury. Estimates of CKD prevalence vary widely, both within and between countries, due to effective differences in CKD regional prevalence, different understandings regarding the use of eGFR for identifying CKD, eGFR thresholds considered to define CKD in elderly populations, analytical methodologies applied for creatinine measurement, formulas for calculation of the eGFR and statistical approaches to estimate CKD prevalence in large-scale epidemiological studies. In an interesting review, solutions to overcome discrepancies were proposed [7]. The KDIGO guidelines [8] are also critical to design epidemiological studies to characterize the global burden of CKD in the general population and subgroups at increased risk with certain comorbidities. In the present work we aimed to study the prevalence of CKD in a population in northern Portugal.

According to the KDIGO guidelines using CKD-EPI and UACR calculations, our results suggest a CKD prevalence of 9.8% for patients in stages ≥G3a/A1. The prevalence of CKD, using two measurements of creatinine clearance calculated by the CG equation and two measurements of UACR, revealed a higher prevalence of 11.3%. The RENA study found a prevalence of CKD stages 1–5 of 20.9% (10.7% for stages ≥G3a) in the Portuguese population that attends the PCHU, while the PREVADIAB study found a prevalence of CKD stages 3–5 of 6.1%, without estimating CKD in stages G1 and G2. As the samples were very similar regarding sex (i.e. 65% of women in the RENA study versus 60% in the PREVADIAB study), age class distribution (i.e. 48% ≥60 years of age in RENA and 46% between 60 and 79 years in PREVADIAB) and comorbidities (i.e. self-reported hypertension, T2DM and obesity in 38%, 16% and 31%, respectively, in the RENA study versus 45%, 12% and 34% in the PREVADIAB study), the different recruitment strategy may partially explain the discrepancies. In fact, in the RENA study the participants were not recruited from the general population, but from primary care attendees, who are possibly less healthy, while in the PREVADIAB study, primarily designed to estimate the prevalence of DM in the Portuguese population, analysed data from a nationally representative sample of 5167 subjects. Therefore the CKD estimation in each study might have introduced some bias compromising full characterization. In our study, the real prevalence was determined from a large and unselected population of 136 993 individuals (121 643 ages 20–79 years), representing 59 867 (43.7%) men and 77 126 (56.3%) women. Comorbidities, such as hypertension and T2DM, with a prevalence of 42.9% (58 698) and 22.9% (31 494), respectively, were highly comparable to the PREVADIAB study, while obesity was less prevalent [*n* = 27 835 (20.3%)] in our study. It is also important to underline that comparisons of CKD prevalence between publications should be made with the due care. Indeed, different equations for eGFR may impact in the estimation of CKD. While the PREVADIAB eGFR was calculated using the simplified (the four-variable formula) Modification of Diet in Renal Disease study equation, in the RENA the CKD-EPI equation was used. In our study, besides the CKD-EPI equation, the CG equation was also used to increase the robustness of our studies.

Compared with other countries, the CKD burden shows marked variations in the prevalence: 3.3% in Norway, 17.3% in northeast Germany [11, 20] and 15.1% in Spain according to the ENRICA study when considering CKD stages 1–5 [21] and more recently estimated to be 4.91% for CKD stages 3–5 [22]. A meta-analysis performed to determine the global prevalence of CKD in 100 studies from all over the world presented a global mean prevalence of CKD stages 1–5 of 13.4% (range 11.7–15.1%) and stages 3–5 of 10.6% (range 9.2–12.2%) [4]. Interestingly, our study also uncovered a higher prevalence of CKD for stages ≥G3a compared with other countries. Indeed, Portugal has one of the highest prevalences in Europe of patients undergoing renal replacement therapy [23]. Nevertheless, another possible explanation is that in Portugal, primary care programs frequently remind patients to visit their family doctor by letter at least once every 3 years, suggesting that early detection may help to diagnosis CKD in the earliest stages [13]. We also emphasize that we were able to identify a significant percentage of patients [27.2% (*n* = 37 292)] with an eGFR of 60–89 ml/min/1.73 m^2^. Although these cases were formally excluded according to the KDIGO guidelines (as they do not have albuminuria as an additional criteria), some of them may represent true cases of CKD. Therefore, in a global screening perspective, revision of these criteria may be useful to reduce underdiagnosis in the earliest stages. Compared with most of the studies in this meta-analysis [4], we also observed a higher prevalence of CKD in females (5.5%) than males (4.2%), a fact that does not corroborate RENA results.

As shown in Tables 1–3, prevalence increased with age, as demonstrated in several previous studies [4, 11, 20, 21]. Nevertheless, these results may be overestimated since eGFR naturally declines with age and the increased prevalence of CKD described in older groups might be due not only to real CKD, but also to normal biological variations in kidney function. Our results also show a higher prevalence of T2DM, structural heart disease, microvascular disease, familial hypercholesterolemia, cardiovascular disease, hypertension, atrial fibrillation, stable and unstable angina, atherosclerotic disease, myocardial infarction, ischaemic and haemorrhagic stroke and heart failure in the CKD population when compared with the population without CKD. In fact, CKD is an increasingly recognized cardiovascular risk factor, associated with greater therapeutic burden, high healthcare costs and reduced life expectancy, as up to half of individuals with CKD die from cardiovascular disease [24–26]. Recently we demonstrated that the coexistence of heart failure and CKD is associated with increased premature mortality, as well as non-fatal cardiovascular events in T2DM patients <65 years old [27, 28]. Moreover, in 2016 the European Guidelines on Cardiovascular Disease Prevention incorporated CKD as a non-traditional cardiovascular disease risk factor, readily identifiable from the analytical measurements of eGFR and UACR, and whose early identification and management may have a significant positive impact on cardiovascular disease prevention [29, 30]. Specifically, they classified individuals with an eGFR <30 ml/min/1.73 m^2^ and diabetic patients with proteinuria as ‘very high risk’ (equivalent to a 10-year predicted risk of cardiovascular mortality ≥10%) and those with an eGFR of 30–59 ml/min/1.73 m^2^ as ‘high risk’ (equivalent to a 10-year predicted risk of cardiovascular mortality of 5–10%).

Of note, 33.8% (*n* = 46 329) of patients were taking renin–angiotensin system blockers (i.e. angiotensin-converting enzyme inhibitors and angiotensin-receptor blockers and angiotensin receptor–neprilysin inhibitors), a fact that is not in line with a recent Spanish study that demonstrate that almost 70% patients were taking these drugs [22, 31].

Our study has some key strengths compared with previous Portuguese CKD prevalence studies, namely, it was not based on an estimation of CKD as in the RENA study [16] and it includes patients >18 years old and without an upper age limit. Moreover, the inclusion of a large and unselected sample offers more robustness to our results, since it is less likely to suffer from non-response bias. As recommended by the KDIGO guidelines [8], evaluation and confirmation of CKD was performed at two different time points at least 3 months apart, in order to fulfil the chronicity criterion and therefore to reduce the possibility of false-positive results, with consequent overestimation of CKD prevalence [32]. Moreover, to increase the accuracy of CKD prevalence estimation and staging, measurements were performed in the same laboratory and by the same method and ICD diagnostic codes were not included. Indeed, it has been demonstrated that ICD diagnostic codes display poor sensitivity and specificity in rapidly identifying progressing CKD patients when compared with the gold standard of eGFR measures, especially due to different practices among health units [33, 34].

There are, however, some limitations to our study. Specifically, our study population was predominantly Caucasian. Therefore the lack of ethnic diversity may restrict the translation of our results to other populations, especially those with substantial genetic differences [35–37]. Indeed, studies have shown that the development of CKD is largely influenced by multiple genetic loci [38]. As >95% of patients were Caucasian, the expected impact of other ethnicities on the overall prevalence estimation is negligible. In addition, since our population is representative of northern Portugal, this may hamper the interpretation and external validity of the results to the rest of the country. Moreover, this is a retrospective study that used secondary data from electronic health records, meaning that measurements such as UACR, fundamental for CKD staging, could not be defined for 77.7% of patients. On the other hand, the higher prevalence of CKD among patients who did have two eGFR or two UACR estimates should be considered an overestimation, as these characteristics may select patients at higher risk of CKD. Missing data may be easily explained since albuminuria, as a biomarker of kidney disease, is usually measured in the primary care setting in patients with comorbidities such as T2DM or hypertension. Therefore this analytical measurement is not widely available in the population free of or at low risk of developing the reported comorbidities and thus may underestimate the prevalence of the first stages of CKD. Finally, our laboratory results may also be influenced by the heterogeneity of techniques and storage conditions used to measure creatinine [39] and albuminuria by immunoassays [40].

## CONCLUSIONS

Estimation of the prevalence of CKD is a key factor guiding healthcare system policies and strategies [41, 42]. In our population, CKD prevalence is estimated to be 9.8%, which is in accordance with the global prevalence of CKD across Europe. Further studies are needed to evaluate if there was a real change in CKD prevalence over a 13-year period between the PREVADIAB study (conducted in 2008), the RENA study (conducted in 2018) and our 2021 study. In a very similar population, recent data suggest that the prevalence of CKD could have changed in the last few years in Spain [22]. The frequency used to screen for UACR presents a considerable variation between high-risk population groups, resulting in a low awareness of CKD as a modifiable risk factor in the no-T2DM population. It is clear that CKD patients must be identified earlier and to develop awareness and educational programs to prevent CKD and its associated diseases, such as T2DM, cardiovascular disease and obesity, to reduce the CKD burden for patients, caregivers and society.

## Supplementary Material

sfac206_Supplemental_File

## Data Availability

All data are incorporated in the article and its online supplementary material.
