# Supplementary material for: Twenty years of real-world data to estimate chronic kidney disease prevalence and staging in an unselected population
Source: Clin Kidney J. 2022 Sep 12;16(1):111–24. doi: 10.1093/ckj/sfac206 (PMC9871850; doi:10.1093/ckj/sfac206)
Supplement: sfac206_Supplemental_File [file sfac206_supplemental_file.docx]

**Supplementary material**

**Table S1** - Detailed characterization of female population accordingly KDIGO guidelines using CKD-EPI. In total, 27097 (35.1%) patients had at least two eGFR assessments. Percentages are presented for the overall female population (n=77126). eGFR (mL/min/1.73 m^2^).

| **N=27094 (35.1%)** | **eGFR ≥ 90**  **235 (0.3%)** | | **eGFR 60-89**  **22072 (28.6%)** | | **eGFR 45-59**  **2510 (3.3%)** | | **eGFR 30-44**  **1403 (1.8%)** | | **eGFR 15-29**  **635 (0.8%)** | | **eGFR < 15**  **242 (0.3%)** | |
| --- | --- | --- | --- | --- | --- | --- | --- | --- | --- | --- | --- | --- |
| **Sociodemographic characteristics** | **n** | **%** | **n** | **%** | **n** | **%** | **n** | **%** | **n** | **%** | **n** | **%** |
| [Age](https://docs.wp3a.careme.mtg.pt/definitions/observations.html#age) (years) | 56.0 (P50) | 13.0 (IQR) | 68.0  (P50) | 20 (IQR) | 81.0 (P50) | 14.0 (IQR) | 83.0 (P50) | 13.0 (IQR) | 85.0 (P50) | 14.0 (IQR) | 83.0 (P50) | 19.0 (IQR) |
| [[20, 79]](https://docs.wp3a.careme.mtg.pt/definitions/observations.html#between-20-and-79) | 228 | 0 | 18339 | 0.2 | 1143 | 0 | 509 | 0 | 205 | 0 | 106 | 0 |
| [Between 50 and 60](https://docs.wp3a.careme.mtg.pt/definitions/observations.html#between-50-and-60) | 88 | 0.1 | 3878 | 4.3 | 86 | 0.1 | 22 | 0 | 11 | 0 | 13 | 0 |
| [Between 60 and 70](https://docs.wp3a.careme.mtg.pt/definitions/observations.html#between-60-and-70) | 71 | 0.1 | 5101 | 5.7 | 344 | 0.4 | 122 | 0.1 | 48 | 0.1 | 38 | 0 |
| [Between 70 and 80](https://docs.wp3a.careme.mtg.pt/definitions/observations.html#between-70-and-80) | 8 | 0 | 6383 | 7.1 | 682 | 0.8 | 356 | 0.4 | 132 | 0.1 | 47 | 0.1 |
| [Aged 80 or](https://docs.wp3a.careme.mtg.pt/definitions/observations.html#aged-80-and-over) more | 4 | 0 | 3727 | 4.2 | 1367 | 1.5 | 894 | 1.0 | 430 | 0.5 | 136 | 0.2 |
| **Body mass index (Kg/m^2^)** | **n** | **%** | **n** | **%** | **n** | **%** | **n** | **%** | **n** | **%** | **n** | **%** |
| [BMI < 18.5](https://docs.wp3a.careme.mtg.pt/definitions/observations.html#less-than-185) | 3 | 0 | 335 | 0.4 | 37 | 0 | 30 | 0 | 15 | 0 | 9 | 0 |
| [BMI [18.5, 25.0[](https://docs.wp3a.careme.mtg.pt/definitions/observations.html#between-185-and-250) | 35 | 0 | 6632 | 7.4 | 590 | 0.7 | 319 | 0.4 | 142 | 0.2 | 55 | 0.1 |
| [BMI [25.0, 30.0[](https://docs.wp3a.careme.mtg.pt/definitions/observations.html#between-250-and-300) | 86 | 0.1 | 8070 | 9.0 | 934 | 1.0 | 503 | 0.6 | 199 | 0.2 | 83 | 0.1 |
| [BMI [30.0, 35.0[](https://docs.wp3a.careme.mtg.pt/definitions/observations.html#between-300-and-350) | 59 | 0.1 | 4370 | 4.9 | 571 | 0.6 | 309 | 0.3 | 147 | 0.2 | 40 | 0 |
| [BMI ≥ 20](https://docs.wp3a.careme.mtg.pt/definitions/observations.html#greater-than-350) | 49 | 0.1 | 1816 | 2.0 | 272 | 0.3 | 175 | 0.2 | 82 | 0.1 | 27 | 0 |
| **Clinical measurements** | **P50** | **IQR** | **P50** | **IQR** | **P50** | **IQR** | **P50** | **IQR** | **P50** | **IQR** | **P50** | **IQR** |
| Weight (Kg) | 74.0 | 21.0 | 67.0 | 16.0 | 68.0 | 18.0 | 68.0 | 18.0 | 69.0 | 19.0 | 66.0 | 21.0 |
| BMI (Kg/m^2^) | 29.7 | 7.4 | 27.1 | 6.7 | 28.0 | 7.1 | 28.3 | 7.1 | 28.3 | 7.7 | 27.9 | 6.8 |
| Waist circumference (cm) | 101.0 | 19.0 | 97.0 | 16.0 | 100 | 16.0 | 101.0 | 17.0 | 103.0 | 16.5 | 104.5 | 16.0 |
| SBP (mmHg) | 136.0 | 22.0 | 133.0 | 20 | 137.0 | 20 | 137.0 | 20 | 135.0 | 24.0 | 137.0 | 24.8 |
| DBP (mmHg) | 84.0 | 11.0 | 79.0 | 13.0 | 76.0 | 14.0 | 75.0 | 14.3 | 74.0 | 15.0 | 73.0 | 18.8 |
| **Echocardiography measurements** | **P50** | **IQR** | **P50** | **IQR** | **P50** | **IQR** | **P50** | **IQR** | **P50** | **IQR** | **P50** | **IQR** |
| Left atrial volume (mL) | 39.0 | 6.0 | 38.0 | 7.0 | 40 | 8.0 | 41.0 | 7.0 | 42.0 | 8.0 | 42.0 | 8.5 |
| Left atrial volume index (mL/m^2^) | 21.7 | 3.6 | 22.3 | 4.4 | 23.5 | 4.8 | 24.0 | 5.1 | 24.6 | 5.4 | 24.6 | 5.8 |
| Left ventricular mass (g) | 123.4 | 24.8 | 126.0 | 35.7 | 136.9 | 39.2 | 138.6 | 39.5 | 142.3 | 42.9 | 152.3 | 50.4 |
| Left ventricular mass index (g/m^2^) | 69.5 | 15.5 | 73.8 | 20.9 | 80.4 | 23.6 | 81.6 | 23.9 | 84.1 | 25.4 | 86.8 | 27.8 |
| Left atrial diameter (mm) | 49.0 | 7.0 | 48.0 | 5.0 | 48.0 | 6.0 | 48.0 | 6.0 | 48.0 | 7.0 | 50 | 7.0 |
| Ejection fraction (%) | 62.5 | 8.3 | 63.0 | 7.0 | 62.0 | 7.0 | 61.0 | 9.0 | 61.0 | 11.0 | 58.0 | 14.0 |
| Left ventricular posterior wall thickness (mL) | 9.0 | 1.0 | 9.0 | 2.0 | 9.0 | 1.0 | 9.0 | 1.0 | 9.0 | 1.0 | 10 | 2.0 |
| Interventricular septum thickness (mm) | 10 | 1.0 | 10 | 2.0 | 10 | 3.0 | 10 | 3.0 | 11.0 | 2.0 | 11.0 | 2.0 |
| **Laboratory measurements** | **P50** | **IQR** | **P50** | **IQR** | **P50** | **IQR** | **P50** | **IQR** | **P50** | **IQR** | **P50** | **IQR** |
| [Hemoglobin (g/dL](https://docs.wp3a.careme.mtg.pt/definitions/measurements.html#hemoglobin)) | 14.0 | 15.6 | 13.9 | 16.7 | 13.4 | 17.2 | 12.5 | 3.3 | 11.6 | 2.6 | 10.5 | 2.5 |
| [Sodium (mEq/L](https://docs.wp3a.careme.mtg.pt/definitions/measurements.html#sodium)) | 140 | 2.0 | 140 | 3.0 | 140 | 4.0 | 140 | 4.0 | 140 | 4.0 | 139.0 | 6.0 |
| [Potassium (mEq/L](https://docs.wp3a.careme.mtg.pt/definitions/measurements.html#potassium) | 4.2 | 0.5 | 4.3 | 0.5 | 4.4 | 0.6 | 4.4 | 0.7 | 4.5 | 0.8 | 4.7 | 1.1 |
| [Phosphate (mg/dL](https://docs.wp3a.careme.mtg.pt/definitions/measurements.html#phosphate)) | 3.4 | 0.8 | 3.4 | 0.7 | 3.4 | 0.8 | 3.4 | 0.8 | 3.7 | 1.0 | 4.2 | 1.7 |
| [Magnesium (mg/dL](https://docs.wp3a.careme.mtg.pt/definitions/measurements.html#magnesium)) | 2.0 | 0.3 | 2.1 | 0.3 | 2.0 | 0.4 | 2.0 | 0.4 | 2.1 | 0.4 | 2.1 | 0.5 |
| [Calcium (mg/dL](https://docs.wp3a.careme.mtg.pt/definitions/measurements.html#calcium)) | 9.3 | 0.6 | 9.4 | 0.6 | 9.4 | 0.7 | 9.4 | 0.7 | 9.3 | 0.8 | 8.9 | 1.1 |
| [Vitamin D (ng/mL](https://docs.wp3a.careme.mtg.pt/definitions/measurements.html#vitamin-d)) | 15.5 | 9.8 | 20 | 14.0 | 17.0 | 16.0 | 14.0 | 18.0 | 18.0 | 20 | 13.0 | 16.5 |
| [Uric acid (µg/dL](https://docs.wp3a.careme.mtg.pt/definitions/measurements.html#uric-acid)) | 4.7 | 1.9 | 4.6 | 1.6 | 5.6 | 2.1 | 6.3 | 2.4 | 7.1 | 3.1 | 7.3 | 3.3 |
| [Creatine Kinase (IU/L](https://docs.wp3a.careme.mtg.pt/definitions/measurements.html#creatine-kinase)) | 67.0 | 67.8 | 78.0 | 59.0 | 76.0 | 63.0 | 71.0 | 66.0 | 73.5 | 72.8 | 68.5 | 72.8 |
| [Iron (µg/L](https://docs.wp3a.careme.mtg.pt/definitions/measurements.html#total-serum-iron)) | 69.0 | 44.8 | 78.0 | 42.0 | 68.0 | 36.0 | 62.0 | 36.0 | 57.0 | 37.0 | 49.0 | 36.0 |
| [Transferrin (µg/L](https://docs.wp3a.careme.mtg.pt/definitions/measurements.html#transferrin)) | 285.0 | 115.0 | 253.0 | 81.0 | 243.0 | 81.0 | 242.0 | 82.5 | 230 | 91.5 | 196.0 | 89.0 |
| [Total iron binding capacity (µg/L](https://docs.wp3a.careme.mtg.pt/definitions/measurements.html#total-iron-binding-capacity)) | 336.0 | 119.0 | 308.0 | 86.0 | 291.0 | 86.4 | 288.0 | 86.0 | 263.0 | 93.0 | 244.0 | 92.0 |
| [Parathyroid hormone (pg/mL](https://docs.wp3a.careme.mtg.pt/definitions/measurements.html#parathyroid-hormone)) | 44.0 | 17.8 | 51.0 | 34.0 | 73.8 | 54.0 | 97.2 | 92.4 | 136.9 | 109.7 | 205.7 | 185.6 |
| [Glucose (mg/dL](https://docs.wp3a.careme.mtg.pt/definitions/measurements.html#glucose)) | 114.0 | 56.3 | 97.0 | 27.0 | 108.0 | 44.0 | 114.0 | 50.6 | 120 | 73.0 | 119.0 | 72.5 |
| [HbA1c (%](https://docs.wp3a.careme.mtg.pt/definitions/measurements.html#glycated-hemoglobin)) | 7.6 | 29.0 | 6.2 | 27.4 | 6.6 | 31.2 | 7.1 | 33.1 | 7.3 | 33.2 | 7.3 | 29.8 |
| [LDL-cholesterol (mg/dL)](https://docs.wp3a.careme.mtg.pt/definitions/measurements.html#cholesterol-ldl) | 118.0 | 49.5 | 121.0 | 44.0 | 112.0 | 44.0 | 104.0 | 43.0 | 101.0 | 45.1 | 105.0 | 49.8 |
| [HDL-cholesterol (mg/dL)](https://docs.wp3a.careme.mtg.pt/definitions/measurements.html#cholesterol-hdl) | 46.0 | 14.5 | 51.0 | 16.0 | 47.0 | 16.0 | 45.0 | 17.0 | 41.0 | 16.0 | 41.0 | 17.0 |
| [Non-HDL-cholesterol (mg/dL](https://docs.wp3a.careme.mtg.pt/definitions/measurements.html#cholesterol-non-hdl)) | 143.0 | 57.0 | 141.0 | 46.0 | 135.0 | 46.0 | 130 | 47.0 | 125.0 | 47.0 | 131.0 | 50.8 |
| [Total cholesterol (mg/dL](https://docs.wp3a.careme.mtg.pt/definitions/measurements.html#cholesterol-total)) | 190 | 59.5 | 195.0 | 48.0 | 184.0 | 49.0 | 177.0 | 51.0 | 170 | 55.5 | 173.0 | 61.0 |
| [Triglycerides (mg/dL)](https://docs.wp3a.careme.mtg.pt/definitions/measurements.html#triglycerides) | 124.0 | 75.5 | 100 | 59.0 | 118.0 | 65.0 | 124.0 | 69.0 | 123.0 | 73.0 | 128.0 | 80.5 |
| Brain natriuretic peptide (pg/mL) | 42.0 | 67.1 | 91.4 | 159.3 | 158.3 | 321.5 | 170.9 | 320.7 | 246.4 | 491.6 | 637.1 | 1143.5 |
| NT-pro-brain natriuretic peptide (pg/mL) | 297 | 5.5 | 826.4 | 2343.8 | 1266.2 | 2816.8 | 2643.7 | 14459.3 | 4020.6 | 4919.4 | 19897.6 | 37531.8 |
| [Albumin (g/dL](https://docs.wp3a.careme.mtg.pt/definitions/measurements.html#albumin)) | 4.3 | 0.7 | 4.4 | 45.6 | 4.3 | 34.7 | 4.2 | 0.9 | 3.9 | 1.0 | 3.4 | 0.9 |
| [Bilirubin (mg/dL](https://docs.wp3a.careme.mtg.pt/definitions/measurements.html#bilirubin)) | 0.5 | 0.3 | 0.6 | 0.3 | 0.6 | 0.4 | 0.5 | 0.3 | 0.5 | 0.3 | 0.5 | 0.4 |
| [ALT (IU/L](https://docs.wp3a.careme.mtg.pt/definitions/measurements.html#alanine-amino-transferase)) | 20 | 12.0 | 17.0 | 9.0 | 16.0 | 10 | 15.0 | 9.5 | 15.0 | 11.0 | 16.0 | 18.0 |
| [AST (IU/L](https://docs.wp3a.careme.mtg.pt/definitions/measurements.html#aspartate-amino-transferase)) | 18.0 | 7.0 | 19.0 | 7.0 | 20 | 8.0 | 20 | 9.0 | 20 | 10 | 21.0 | 15.5 |
| [Alkaline phosphatase (IU/L](https://docs.wp3a.careme.mtg.pt/definitions/measurements.html#alkaline-phosphatase)) | 73.5 | 36.3 | 72.0 | 30 | 77.0 | 36.0 | 82.0 | 40.8 | 91.0 | 51.5 | 95.0 | 56.3 |
| [TSH (IU/mL](https://docs.wp3a.careme.mtg.pt/definitions/measurements.html#thyroid-stimulating-hormone)) | 1.6 | 1.1 | 1.7 | 1.3 | 1.8 | 1.5 | 1.8 | 1.6 | 1.8 | 1.8 | 1.7 | 1.6 |
| [T3 (µg/dL](https://docs.wp3a.careme.mtg.pt/definitions/measurements.html#triiodothyronine)) | 2.8 | 0.7 | 2.8 | 0.6 | 2.7 | 0.7 | 2.6 | 0.7 | 2.5 | 0.9 | 2.4 | 0.8 |
| [T4 (µg/dL](https://docs.wp3a.careme.mtg.pt/definitions/measurements.html#thyroxine)) | 1.0 | 0.2 | 1.0 | 0.2 | 1.0 | 0.2 | 1.0 | 0.2 | 1.0 | 0.2 | 1.0 | 0.3 |
| [Creatinine (mg/dL](https://docs.wp3a.careme.mtg.pt/definitions/measurements.html#creatinine)) | 0.7 | 0.1 | 0.8 | 0.1 | 1.0 | 0.2 | 1.3 | 0.2 | 1.9 | 0.5 | 3.8 | 1.1 |
| [UACR (mg/g](https://docs.wp3a.careme.mtg.pt/definitions/measurements.html#urinary-albumin-creatinine-ratio)) | 77.5 | 128.8 | 9.0 | 13.7 | 13.2 | 28.6 | 19.4 | 54.4 | 40.5 | 151.9 | 228.4 | 1091.2 |
| **Comorbidities** | **n** | **%** | **n** | **%** | **n** | **%** | **n** | **%** | **n** | **%** | **n** | **%** |
| [Obesity](https://docs.wp3a.careme.mtg.pt/definitions/comorbidities.html#obesity) | 108 | 0.1 | 6186 | 6.9 | 843 | 0.9 | 484 | 0.5 | 229 | 0.3 | 67 | 0.1 |
| [Hypercholesterolemia](https://docs.wp3a.careme.mtg.pt/definitions/comorbidities.html#hypercholesterolemia) | 117 | 0.1 | 11910 | 13.3 | 1041 | 1.2 | 484 | 0.5 | 190 | 0.2 | 89 | 0.1 |
| [Type 2 diabetes](https://docs.wp3a.careme.mtg.pt/definitions/comorbidities.html#diabetes-mellitus-type-2) mellitus | 152 | 0.2 | 7248 | 8.1 | 1299 | 1.5 | 903 | 1.0 | 467 | 0.5 | 181 | 0.2 |
| [Structural heart disease](https://docs.wp3a.careme.mtg.pt/definitions/comorbidities.html#heart-disease-structural) | 48 | 0.1 | 3466 | 3.9 | 786 | 0.9 | 618 | 0.7 | 380 | 0.4 | 169 | 0.2 |
| [Microvascular disease](https://docs.wp3a.careme.mtg.pt/definitions/comorbidities.html#microvascular-disease) | 37 | 0 | 1403 | 1.6 | 262 | 0.3 | 240 | 0.3 | 168 | 0.2 | 91 | 0.1 |
| [Cardiovascular disease](https://docs.wp3a.careme.mtg.pt/definitions/comorbidities.html#cardiovascular-disease) | 218 | 0.2 | 15844 | 17.7 | 2510 | 2.8 | 1403 | 1.6 | 635 | 0.7 | 242 | 0.3 |
| [Hypertension](https://docs.wp3a.careme.mtg.pt/definitions/comorbidities.html#hypertension) | 210 | 0.2 | 14891 | 16.7 | 2273 | 2.5 | 1304 | 1.5 | 582 | 0.7 | 215 | 0.2 |
| [Atrial fibrillation](https://docs.wp3a.careme.mtg.pt/definitions/comorbidities.html#atrial-fibrillation) | 9 | 0 | 1261 | 1.4 | 437 | 0.5 | 314 | 0.4 | 217 | 0.2 | 81 | 0.1 |
| [Stable angina](https://docs.wp3a.careme.mtg.pt/definitions/comorbidities.html#angina-stable) | 3 | 0 | 534 | 0.6 | 163 | 0.2 | 138 | 0.2 | 79 | 0.1 | 50 | 0.1 |
| [Transient ischemic attack](https://docs.wp3a.careme.mtg.pt/definitions/comorbidities.html#transient-ischemic-attack) | 3 | 0 | 201 | 0.2 | 53 | 0.1 | 33 | 0 | 23 | 0 | 8 | 0 |
| [Atherosclerotic disease](https://docs.wp3a.careme.mtg.pt/definitions/comorbidities.html#arterosclerotic-disease) | 30 | 0 | 2350 | 2.6 | 569 | 0.6 | 400 | 0.4 | 270 | 0.3 | 115 | 0.1 |
| [Unstable angina](https://docs.wp3a.careme.mtg.pt/definitions/comorbidities.html#angina-unstable) | 2 | 0 | 282 | 0.3 | 97 | 0.1 | 70 | 0.1 | 36 | 0 | 19 | 0 |
| [Myocardial](https://docs.wp3a.careme.mtg.pt/definitions/comorbidities.html#myocardial-infarction) infarction | 4 | 0 | 360 | 0.4 | 121 | 0.1 | 96 | 0.1 | 77 | 0.1 | 49 | 0.1 |
| [Stroke](https://docs.wp3a.careme.mtg.pt/definitions/comorbidities.html#stroke) | 24 | 0 | 1750 | 2.0 | 386 | 0.4 | 262 | 0.3 | 170 | 0.2 | 72 | 0.1 |
| [Ischemic stroke](https://docs.wp3a.careme.mtg.pt/definitions/comorbidities.html#ischemic) | 19 | 0 | 1426 | 1.6 | 322 | 0.4 | 229 | 0.3 | 152 | 0.2 | 65 | 0.1 |
| [Hemorrhagic](https://docs.wp3a.careme.mtg.pt/definitions/comorbidities.html#hemorrhagic) stroke | 2 | 0 | 132 | 0.1 | 25 | 0 | 18 | 0 | 12 | 0 | 10 | 0 |
| [Peripheral artery disease](https://docs.wp3a.careme.mtg.pt/definitions/comorbidities.html#peripheral-artery-disease) | 9 | 0 | 618 | 0.7 | 175 | 0.2 | 117 | 0.1 | 77 | 0.1 | 38 | 0 |
| [Heart failure](https://docs.wp3a.careme.mtg.pt/definitions/comorbidities.html#stage-latest) | 2 | 0 | 489 | 0.5 | 234 | 0.3 | 211 | 0.2 | 177 | 0.2 | 91 | 0.1 |
| [Preserved](https://docs.wp3a.careme.mtg.pt/definitions/comorbidities.html#stage-latest) | 2 | 0 | 432 | 0.5 | 201 | 0.2 | 173 | 0.2 | 145 | 0.2 | 70 | 0.1 |
| [Midrange](https://docs.wp3a.careme.mtg.pt/definitions/comorbidities.html#stage-latest) | 0 | 0 | 55 | 0.1 | 32 | 0 | 35 | 0 | 25 | 0 | 17 | 0 |
| [Reduced](https://docs.wp3a.careme.mtg.pt/definitions/comorbidities.html#stage-latest) | 0 | 0 | 2 | 0 | 1 | 0 | 3 | 0 | 7 | 0 | 4 | 0 |
| **Cardiovascular medications** | **n** | **%** | **n** | **%** | **n** | **%** | **n** | **%** | **n** | **%** | **n** | **%** |
| Renin-angiotensin-system-acting agents | 198 | 0.2 | 12186 | 13.6 | 2149 | 2.4 | 1274 | 1.4 | 574 | 0.6 | 221 | 0.2 |
| Angiotensin-converting enzyme inhibitors | 155 | 0.2 | 8305 | 9.3 | 1555 | 1.7 | 954 | 1.1 | 431 | 0.5 | 165 | 0.2 |
| Angiotensin receptor blockers | 123 | 0.1 | 7896 | 8.8 | 1467 | 1.6 | 916 | 1.0 | 416 | 0.5 | 165 | 0.2 |
| Angiotensin receptor-neprilysin inhibitors | 0 | 0 | 14 | 0 | 8 | 0 | 7 | 0 | 1 | 0 | 0 | 0 |
| Diuretics | 104 | 0.1 | 7562 | 8.5 | 1541 | 1.7 | 1055 | 1.2 | 538 | 0.6 | 205 | 0.2 |
| Thiazides | 3 | 0 | 116 | 0.1 | 21 | 0 | 11 | 0 | 5 | 0 | 1 | 0 |
| Sulfonamides | 55 | 0.1 | 3870 | 4.3 | 1026 | 1.1 | 847 | 0.9 | 487 | 0.5 | 197 | 0.2 |
| Aldosterone antagonists | 10 | 0 | 785 | 0.9 | 187 | 0.2 | 169 | 0.2 | 103 | 0.1 | 34 | 0 |
| Antiplatelets | 69 | 0.1 | 5115 | 5.7 | 1140 | 1.3 | 747 | 0.8 | 361 | 0.4 | 154 | 0.2 |
| Low dose Aspirin® | 67 | 0.1 | 4614 | 5.2 | 1031 | 1.2 | 683 | 0.8 | 329 | 0.4 | 144 | 0.2 |
| P2y12 antagonists | 11 | 0 | 1218 | 1.4 | 308 | 0.3 | 220 | 0.2 | 109 | 0.1 | 49 | 0.1 |
| Anticoagulants | 15 | 0 | 1610 | 1.8 | 458 | 0.5 | 327 | 0.4 | 178 | 0.2 | 60 | 0.1 |
| Vitamin K antagonists | 14 | 0 | 822 | 0.9 | 276 | 0.3 | 204 | 0.2 | 125 | 0.1 | 50 | 0.1 |
| Novel oral anticoagulants | 5 | 0 | 983 | 1.1 | 259 | 0.3 | 180 | 0.2 | 93 | 0.1 | 12 | 0 |
| Calcium channel blockers | 79 | 0.1 | 4390 | 4.9 | 1098 | 1.2 | 777 | 0.9 | 394 | 0.4 | 169 | 0.2 |
| Beta blockers | 92 | 0.1 | 5889 | 6.6 | 1109 | 1.2 | 725 | 0.8 | 363 | 0.4 | 144 | 0.2 |
| Nitrates | 5 | 0 | 897 | 1.0 | 267 | 0.3 | 221 | 0.2 | 126 | 0.1 | 63 | 0.1 |
| **Diabetes medications** | **n** | **%** | **n** | **%** | **n** | **%** | **n** | **%** | **n** | **%** | **n** | **%** |
| Glucose lowering drugs | 129 | 0.1 | 5044 | 5.6 | 943 | 1.1 | 644 | 0.7 | 325 | 0.4 | 126 | 0.1 |
| Excluding insulins | 122 | 0.1 | 4980 | 5.6 | 934 | 1.0 | 635 | 0.7 | 314 | 0.4 | 121 | 0.1 |
| Biguanides | 116 | 0.1 | 4647 | 5.2 | 871 | 1.0 | 581 | 0.6 | 279 | 0.3 | 107 | 0.1 |
| Sodium-glucose co-transporter-2 inhibitors | 29 | 0 | 461 | 0.5 | 99 | 0.1 | 44 | 0 | 15 | 0 | 0 | 0 |
| Glucagon-like peptide-1 receptor agonists | 20 | 0 | 196 | 0.2 | 43 | 0 | 33 | 0 | 17 | 0 | 1 | 0 |
| Dipeptidyl peptidase-4 inhibitors | 71 | 0.1 | 1915 | 2.1 | 525 | 0.6 | 416 | 0.5 | 228 | 0.3 | 87 | 0.1 |
| Glitazones | 16 | 0 | 394 | 0.4 | 125 | 0.1 | 109 | 0.1 | 58 | 0.1 | 25 | 0 |
| Sulfonylureas | 56 | 0.1 | 1735 | 1.9 | 486 | 0.5 | 344 | 0.4 | 180 | 0.2 | 75 | 0.1 |
| Metiglinides | 2 | 0 | 63 | 0.1 | 15 | 0 | 11 | 0 | 10 | 0 | 4 | 0 |
| Glucosidase inhibitors | 13 | 0 | 332 | 0.4 | 109 | 0.1 | 85 | 0.1 | 42 | 0 | 28 | 0 |
| Insulins | 48 | 0.1 | 708 | 0.8 | 213 | 0.2 | 227 | 0.3 | 154 | 0.2 | 86 | 0.1 |
| Long acting | 33 | 0 | 460 | 0.5 | 143 | 0.2 | 162 | 0.2 | 111 | 0.1 | 52 | 0.1 |
| Intermediate acting | 20 | 0 | 336 | 0.4 | 92 | 0.1 | 101 | 0.1 | 79 | 0.1 | 49 | 0.1 |
| Fast acting | 16 | 0 | 208 | 0.2 | 53 | 0.1 | 59 | 0.1 | 50 | 0.1 | 27 | 0 |
| Premixed | 15 | 0 | 213 | 0.2 | 69 | 0.1 | 68 | 0.1 | 39 | 0 | 28 | 0 |
| **Bone medications** | **n** | **%** | **n** | **%** | **n** | **%** | **n** | **%** | **n** | **%** | **n** | **%** |
| [Bone Disease Medications](https://docs.wp3a.careme.mtg.pt/definitions/medications.html#bone-disease-medication) | 18 | 0 | 5885 | 6.6 | 870 | 1.0 | 443 | 0.5 | 175 | 0.2 | 39 | 0 |
| [Vitamin D](https://docs.wp3a.careme.mtg.pt/definitions/medications.html#vitamin-d-and-analogues) | 30 | 0 | 3646 | 4.1 | 521 | 0.6 | 373 | 0.4 | 231 | 0.3 | 79 | 0.1 |
| [Calcium](https://docs.wp3a.careme.mtg.pt/definitions/medications.html#calcium) | 30 | 0 | 5726 | 6.4 | 798 | 0.9 | 411 | 0.5 | 182 | 0.2 | 58 | 0.1 |
| [Magnesium](https://docs.wp3a.careme.mtg.pt/definitions/medications.html#magnesium) | 34 | 0 | 4247 | 4.7 | 571 | 0.6 | 338 | 0.4 | 150 | 0.2 | 54 | 0.1 |
| [Estrogens and progestogens](https://docs.wp3a.careme.mtg.pt/definitions/medications.html#estrogens-and-progestogens) | 0 | 0 | 0 | 0 | 0 | 0 | 0 | 0 | 0 | 0 | 0 | 0 |
| [Calcitonins](https://docs.wp3a.careme.mtg.pt/definitions/medications.html#calcitonins) | 70 | 0.1 | 8136 | 9.1 | 773 | 0.9 | 379 | 0.4 | 147 | 0.2 | 37 | 0 |

**Table S2** - Detailed characterization of male population accordingly KDIGO guidelines using CKD-EPI. In total, 18886 (31.5%) patients had at least two eGFR assessments. Percentages are presented for the overall male population (n=59867). eGFR (mL/min/1.73 m^2^).

| **n=18886 (31.5%)** | **eGFR ≥ 90**  **417 (0.7%)** | | **eGFR 60-89**  **15220 (25.4%)** | | **eGFR 45-59**  **1812 (3.0%)** | | **eGFR 30-44**  **873 (1.5%)** | | **eGFR 15-29**  **403 (0.7%)** | | **eGFR < 15**  **161 (0.3%)** | |
| --- | --- | --- | --- | --- | --- | --- | --- | --- | --- | --- | --- | --- |
| **Sociodemographic characteristics** | **n** | **%** | **n** | **%** | **n** | **%** | **n** | **%** | **n** | **%** | **n** | **%** |
| [Age](https://docs.wp3a.careme.mtg.pt/definitions/observations.html#age) (years) | 58.0 (P50) | 12.3 (IRQ) | 68.0 (P50) | 19.0 (IRQ) | 76.0 (P50) | 13.0 (IRQ) | 79.0 (P50) | 15.0 (IRQ) | 81.0 (P50) | 16.0 (IRQ) | 77.0 (P50) | 15.0 (IRQ) |
| [[20, 79]](https://docs.wp3a.careme.mtg.pt/definitions/observations.html#between-20-and-79) | 414 | 98.6 | 12851 | 84.4 | 1166 | 64.3 | 453 | 51.9 | 189 | 46.9 | 91 | 56.5 |
| [Between 50 and 60](https://docs.wp3a.careme.mtg.pt/definitions/observations.html#between-50-and-60) | 154 | 36.7 | 2 270 | 14.9 | 80 | 4.4 | 35 | 4.0 | 19 | 4.7 | 13 | 8.1 |
| [Between 60 and 70](https://docs.wp3a.careme.mtg.pt/definitions/observations.html#between-60-and-70) | 151 | 36.0 | 3 607 | 23.7 | 328 | 18.1 | 128 | 14.7 | 57 | 14.1 | 25 | 15.5 |
| [Between 70 and 80](https://docs.wp3a.careme.mtg.pt/definitions/observations.html#between-70-and-80) | 29 | 6.9 | 4 584 | 30.1 | 721 | 39.8 | 282 | 32.3 | 101 | 25.1 | 48 | 29.8 |
| [Aged 80 or](https://docs.wp3a.careme.mtg.pt/definitions/observations.html#aged-80-and-over) more | 3 | 0.7 | 2 362 | 15.5 | 646 | 35.6 | 420 | 48.1 | 214 | 53.1 | 70 | 43.5 |
| **Body mass index (Kg/m^2^)** | **n** | **%** | **n** | **%** | **n** | **%** | **n** | **%** | **n** | **%** | **n** | **%** |
| [BMI < 18.5](https://docs.wp3a.careme.mtg.pt/definitions/observations.html#less-than-185) | 4 | 1.0 | 123 | 0.8 | 20 | 1.1 | 10 | 1.1 | 6 | 1.5 | 1 | 0.6 |
| [BMI [18.5, 25.0[](https://docs.wp3a.careme.mtg.pt/definitions/observations.html#between-185-and-250) | 69 | 16.4 | 4 131 | 27.1 | 502 | 27.7 | 221 | 25.3 | 118 | 29.3 | 59 | 36.6 |
| [BMI [25.0, 30.0[](https://docs.wp3a.careme.mtg.pt/definitions/observations.html#between-250-and-300) | 156 | 37.1 | 7 015 | 46.1 | 800 | 44.1 | 381 | 43.6 | 161 | 40 | 64 | 39.8 |
| [BMI [30.0, 35.0[](https://docs.wp3a.careme.mtg.pt/definitions/observations.html#between-300-and-350) | 119 | 28.3 | 2 557 | 16.8 | 342 | 18.9 | 173 | 19.8 | 75 | 18.6 | 25 | 15.5 |
| [BMI ≥ 20](https://docs.wp3a.careme.mtg.pt/definitions/observations.html#greater-than-350) | 62 | 14.8 | 559 | 3.7 | 78 | 4.3 | 46 | 5.3 | 20 | 5.0 | 1 | 0.6 |
| **Clinical measurements** | **P50** | **IQR** | **P50** | **IQR** | **P50** | **IQR** | **P50** | **IQR** | **P50** | **IQR** | **P50** | **IQR** |
| Weight (Kg) | 83.0 | 21.0 | 78.0 | 17.0 | 77.0 | 16.0 | 77.0 | 16.0 | 76.0 | 19.0 | 73.0 | 17.0 |
| BMI (Kg/m^2^) | 29.1 | 6.8 | 26.9 | 4.9 | 26.9 | 5.2 | 27.4 | 5.4 | 26.7 | 5.6 | 26.1 | 5.4 |
| Waist circumference (cm) | 103.0 | 16.0 | 100 | 14.0 | 102.0 | 15.0 | 103.5 | 16.0 | 104.0 | 17.0 | 102.0 | 15.0 |
| SBP (mmHg) | 139.0 | 17.0 | 135.0 | 17.0 | 136.0 | 18.0 | 136.0 | 21.0 | 138.0 | 24.8 | 139.0 | 24.5 |
| DBP (mmHg) | 83.0 | 13.0 | 80 | 12.0 | 76.0 | 13.0 | 75.0 | 15.0 | 73.5 | 16.0 | 72.0 | 18.0 |
| **Echocardiography measurements** | **P50** | **IQR** | **P50** | **IQR** | **P50** | **IQR** | **P50** | **IQR** | **P50** | **IQR** | **P50** | **IQR** |
| Left atrial volume (mL) | 41.0 | 6.0 | 40 | 7.0 | 41.0 | 7.0 | 42.0 | 7.0 | 43.0 | 8.0 | 43.0 | 8.0 |
| Left atrial volume index (mL/m^2^) | 20.9 | 3.5 | 21.4 | 3.8 | 22.0 | 4.3 | 22.6 | 4.6 | 23.0 | 5.1 | 23.3 | 5.1 |
| Left ventricular mass (g) | 151.2 | 41.2 | 145.3 | 40.6 | 151.3 | 49.7 | 156.9 | 49.0 | 160.3 | 58.4 | 163.3 | 59.0 |
| Left ventricular mass index (g/m^2^) | 77.0 | 20 | 77.5 | 21.4 | 82.2 | 25.0 | 82.6 | 28.4 | 87.7 | 29.6 | 89.3 | 30.4 |
| Left atrial diameter (mm) | 51.0 | 6.5 | 51.0 | 6.0 | 51.0 | 7.0 | 51.0 | 7.0 | 52.0 | 7.0 | 52.0 | 7.0 |
| Ejection fraction (%) | 60 | 8.0 | 61.0 | 8.0 | 60 | 11.0 | 60 | 12.0 | 59.0 | 14.0 | 59.0 | 10.8 |
| Left ventricular posterior wall thickness (mL) | 9.0 | 1.0 | 9.0 | 1.0 | 10 | 1.0 | 10 | 2.0 | 10 | 2.0 | 10 | 2.0 |
| Interventricular septum thickness (mm) | 11.0 | 2.0 | 10 | 2.0 | 11.0 | 2.0 | 11.0 | 2.0 | 11.0 | 2.0 | 11.0 | 3.0 |
| **Laboratory measurements** | **P50** | **IQR** | **P50** | **IQR** | **P50** | **IQR** | **P50** | **IQR** | **P50** | **IQR** | **P50** | **IQR** |
| [Hemoglobin (g/dL](https://docs.wp3a.careme.mtg.pt/definitions/measurements.html#hemoglobin)) | 15.5 | 16.2 | 15.4 | 15.9 | 14.6 | 15.4 | 13.7 | 3.5 | 12.1 | 3.1 | 10.9 | 2.4 |
| [Sodium (mEq/L](https://docs.wp3a.careme.mtg.pt/definitions/measurements.html#sodium)) | 139.0 | 4.0 | 140 | 3.0 | 140 | 3.0 | 140 | 4.0 | 140 | 4.0 | 138.0 | 6.0 |
| [Potassium (mEq/L](https://docs.wp3a.careme.mtg.pt/definitions/measurements.html#potassium) | 4.3 | 0.5 | 4.3 | 0.5 | 4.4 | 0.6 | 4.5 | 0.7 | 4.6 | 0.9 | 4.7 | 1.2 |
| [Phosphate (mg/dL](https://docs.wp3a.careme.mtg.pt/definitions/measurements.html#phosphate)) | 3.3 | 1.0 | 3.2 | 0.7 | 3.2 | 0.7 | 3.2 | 0.9 | 3.5 | 0.9 | 4.4 | 1.8 |
| [Magnesium (mg/dL](https://docs.wp3a.careme.mtg.pt/definitions/measurements.html#magnesium)) | 2.0 | 0.3 | 2.1 | 0.3 | 2.0 | 0.3 | 2.0 | 0.4 | 2.1 | 0.4 | 2.2 | 0.4 |
| [Calcium (mg/dL](https://docs.wp3a.careme.mtg.pt/definitions/measurements.html#calcium)) | 9.6 | 0.6 | 9.4 | 0.7 | 9.4 | 0.7 | 9.4 | 0.8 | 9.3 | 0.8 | 8.9 | 0.9 |
| [Vitamin D (ng/mL](https://docs.wp3a.careme.mtg.pt/definitions/measurements.html#vitamin-d)) | 18.0 | 14.0 | 19.0 | 15.0 | 16.0 | 14.8 | 18.0 | 16.0 | 20 | 19.0 | 18.0 | 15.0 |
| [Uric acid (µg/dL](https://docs.wp3a.careme.mtg.pt/definitions/measurements.html#uric-acid)) | 5.9 | 1.9 | 5.9 | 1.9 | 6.6 | 2.3 | 6.9 | 2.5 | 7.4 | 2.8 | 7.2 | 2.5 |
| [Creatine Kinase (IU/L](https://docs.wp3a.careme.mtg.pt/definitions/measurements.html#creatine-kinase)) | 101.5 | 101.5 | 97.0 | 88.0 | 83.0 | 80 | 84.0 | 79.0 | 78.5 | 83.8 | 91.0 | 106.0 |
| [Iron (µg/L](https://docs.wp3a.careme.mtg.pt/definitions/measurements.html#total-serum-iron)) | 74.5 | 56.0 | 87.0 | 50 | 77.0 | 49.0 | 72.0 | 46.0 | 62.0 | 41.0 | 50.5 | 44.0 |
| [Transferrin (µg/L](https://docs.wp3a.careme.mtg.pt/definitions/measurements.html#transferrin)) | 231.0 | 67.0 | 241.0 | 66.3 | 232.0 | 80.3 | 229.0 | 69.5 | 217.0 | 82.3 | 199.0 | 68.0 |
| [Total iron binding capacity (µg/L](https://docs.wp3a.careme.mtg.pt/definitions/measurements.html#total-iron-binding-capacity)) | 298.0 | 94.0 | 292.6 | 75.0 | 286.0 | 82.5 | 279.0 | 80 | 262.0 | 81.3 | 237.5 | 77.3 |
| [Parathyroid hormone (pg/mL](https://docs.wp3a.careme.mtg.pt/definitions/measurements.html#parathyroid-hormone)) | 28.9 | 19.4 | 48.0 | 37.6 | 68.7 | 47.2 | 87.2 | 59.9 | 124.7 | 113.8 | 193.3 | 228.7 |
| [Glucose (mg/dL](https://docs.wp3a.careme.mtg.pt/definitions/measurements.html#glucose)) | 128.4 | 62.7 | 103.0 | 30.6 | 109.0 | 42.0 | 116.9 | 53.1 | 117.0 | 57.0 | 118.0 | 60 |
| [HbA1c (%](https://docs.wp3a.careme.mtg.pt/definitions/measurements.html#glycated-hemoglobin)) | 8.0 | 37.1 | 6.4 | 28.4 | 6.5 | 30.2 | 7.3 | 32.2 | 7.4 | 31.2 | 7.3 | 31.2 |
| [LDL-cholesterol (mg/dL)](https://docs.wp3a.careme.mtg.pt/definitions/measurements.html#cholesterol-ldl) | 109.0 | 46.0 | 115.0 | 45.0 | 102.0 | 43.0 | 97.0 | 48.0 | 91.5 | 46.7 | 96.5 | 49.3 |
| [HDL-cholesterol (mg/dL)](https://docs.wp3a.careme.mtg.pt/definitions/measurements.html#cholesterol-hdl) | 42.0 | 14.0 | 44.0 | 15.0 | 41.0 | 14.0 | 39.0 | 15.0 | 37.0 | 14.3 | 39.0 | 16.0 |
| [Non-HDL-cholesterol (mg/dL](https://docs.wp3a.careme.mtg.pt/definitions/measurements.html#cholesterol-non-hdl)) | 137.0 | 57.0 | 136.6 | 50 | 126.0 | 47.0 | 122.0 | 51.0 | 116.0 | 44.0 | 116.0 | 45.0 |
| [Total cholesterol (mg/dL](https://docs.wp3a.careme.mtg.pt/definitions/measurements.html#cholesterol-total)) | 184.0 | 56.0 | 183.0 | 53.0 | 169.0 | 51.0 | 166.0 | 52.0 | 158.0 | 52.0 | 157.0 | 49.0 |
| [Triglycerides (mg/dL)](https://docs.wp3a.careme.mtg.pt/definitions/measurements.html#triglycerides) | 130 | 85.5 | 107.0 | 66.0 | 113.0 | 71.0 | 121.0 | 75.0 | 121.0 | 69.0 | 113.0 | 69.0 |
| Brain natriuretic peptide (pg/mL) | 62.0 | 156.5 | 90.5 | 161.5 | 146.5 | 276.6 | 146.4 | 331.3 | 241.3 | 466.3 | 660.2 | 1052.1 |
| NT-pro-brain natriuretic peptide (pg/mL) | 303.1 | 0 | 663.3 | 1943.8 | 2661.6 | 5528.0 | 4148.2 | 6399.1 | 5504.3 | 10457.6 | 13872.3 | 15405.4 |
| [Albumin (g/dL](https://docs.wp3a.careme.mtg.pt/definitions/measurements.html#albumin)) | 4.5 | 0.8 | 4.5 | 39.7 | 4.4 | 0.9 | 4.2 | 0.8 | 4.0 | 1.0 | 3.6 | 0.8 |
| [Bilirubin (mg/dL](https://docs.wp3a.careme.mtg.pt/definitions/measurements.html#bilirubin)) | 0.6 | 0.4 | 0.7 | 0.5 | 0.7 | 0.4 | 0.6 | 0.4 | 0.6 | 0.5 | 0.5 | 0.5 |
| [ALT (IU/L](https://docs.wp3a.careme.mtg.pt/definitions/measurements.html#alanine-amino-transferase)) | 28.0 | 20 | 22.0 | 14.0 | 19.0 | 12.0 | 18.0 | 12.0 | 18.0 | 13.0 | 17.0 | 15.0 |
| [AST (IU/L](https://docs.wp3a.careme.mtg.pt/definitions/measurements.html#aspartate-amino-transferase)) | 23.0 | 12.0 | 21.0 | 8.0 | 20 | 9.0 | 21.0 | 10 | 21.0 | 11.0 | 21.0 | 18.0 |
| [Alkaline phosphatase (IU/L](https://docs.wp3a.careme.mtg.pt/definitions/measurements.html#alkaline-phosphatase)) | 73.0 | 28.0 | 68.0 | 27.0 | 71.0 | 33.0 | 75.0 | 35.0 | 88.0 | 51.0 | 91.0 | 57.0 |
| [TSH (IU/mL](https://docs.wp3a.careme.mtg.pt/definitions/measurements.html#thyroid-stimulating-hormone)) | 1.5 | 1.0 | 1.5 | 1.1 | 1.6 | 1.2 | 1.6 | 1.2 | 1.7 | 1.6 | 1.6 | 1.1 |
| [T3 (µg/dL](https://docs.wp3a.careme.mtg.pt/definitions/measurements.html#triiodothyronine)) | 3.0 | 0.7 | 2.9 | 0.7 | 2.7 | 0.8 | 2.7 | 0.7 | 2.5 | 0.7 | 2.4 | 0.8 |
| [T4 (µg/dL](https://docs.wp3a.careme.mtg.pt/definitions/measurements.html#thyroxine)) | 1.0 | 0.2 | 1.0 | 0.2 | 1.0 | 0.2 | 1.0 | 0.2 | 1.0 | 0.2 | 1.0 | 0.3 |
| [Creatinine (mg/dL](https://docs.wp3a.careme.mtg.pt/definitions/measurements.html#creatinine)) | 0.8 | 0.2 | 1.0 | 0.2 | 1.3 | 0.2 | 1.7 | 0.3 | 2.5 | 0.7 | 4.5 | 0.7 |
| [UACR (mg/g](https://docs.wp3a.careme.mtg.pt/definitions/measurements.html#urinary-albumin-creatinine-ratio)) | 85.8 | 135.0 | 8.7 | 13.6 | 13.8 | 37.7 | 26.4 | 135.1 | 102.5 | 497.0 | 902.8 | 1979.4 |
| **Comorbidities** | **n** | **%** | **n** | **%** | **n** | **%** | **n** | **%** | **n** | **%** | **n** | **%** |
| [Obesity](https://docs.wp3a.careme.mtg.pt/definitions/comorbidities.html#obesity) | 181 | 43.1 | 3 116 | 20.5 | 420 | 23.2 | 219 | 25.1 | 95 | 23.6 | 26 | 16.1 |
| [Hypercholesterolemia](https://docs.wp3a.careme.mtg.pt/definitions/comorbidities.html#hypercholesterolemia) | 175 | 41.7 | 6 208 | 40.8 | 512 | 28.2 | 220 | 25.2 | 79 | 19.6 | 30 | 18.6 |
| [Type 2 diabetes](https://docs.wp3a.careme.mtg.pt/definitions/comorbidities.html#diabetes-mellitus-type-2) mellitus | 309 | 73.6 | 5 740 | 37.7 | 1 004 | 55.4 | 579 | 66.3 | 294 | 73.0 | 125 | 77.6 |
| [Structural heart disease](https://docs.wp3a.careme.mtg.pt/definitions/comorbidities.html#heart-disease-structural) | 94 | 22.4 | 2 985 | 19.6 | 652 | 36.0 | 429 | 49.1 | 266 | 66.0 | 129 | 80.1 |
| [Microvascular disease](https://docs.wp3a.careme.mtg.pt/definitions/comorbidities.html#microvascular-disease) | 43 | 10.2 | 521 | 3.4 | 172 | 9.5 | 166 | 19.0 | 108 | 26.8 | 67 | 41.6 |
| [Cardiovascular disease](https://docs.wp3a.careme.mtg.pt/definitions/comorbidities.html#cardiovascular-disease) | 390 | 92.9 | 11311 | 74.3 | 1 813 | 100 | 873 | 100 | 403 | 100 | 161 | 100 |
| [Hypertension](https://docs.wp3a.careme.mtg.pt/definitions/comorbidities.html#hypertension) | 385 | 91.7 | 10540 | 69.2 | 1 608 | 88.7 | 796 | 91.2 | 356 | 88.3 | 141 | 87.6 |
| [Atrial fibrillation](https://docs.wp3a.careme.mtg.pt/definitions/comorbidities.html#atrial-fibrillation) | 16 | 3.8 | 1095 | 7.2 | 302 | 16.7 | 189 | 21.6 | 128 | 31.8 | 42 | 26.1 |
| [Stable angina](https://docs.wp3a.careme.mtg.pt/definitions/comorbidities.html#angina-stable) | 42 | 10 | 1053 | 6.9 | 233 | 12.9 | 157 | 18.0 | 97 | 24.1 | 47 | 29.2 |
| [Transient ischemic attack](https://docs.wp3a.careme.mtg.pt/definitions/comorbidities.html#transient-ischemic-attack) | 8 | 1.9 | 205 | 1.3 | 36 | 2.0 | 30 | 3.4 | 15 | 3.7 | 4 | 2.5 |
| [Atherosclerotic disease](https://docs.wp3a.careme.mtg.pt/definitions/comorbidities.html#arterosclerotic-disease) | 96 | 22.9 | 2508 | 16.5 | 573 | 31.6 | 353 | 40.4 | 182 | 45.2 | 89 | 55.3 |
| [Unstable angina](https://docs.wp3a.careme.mtg.pt/definitions/comorbidities.html#angina-unstable) | 18 | 4.3 | 526 | 3.5 | 116 | 6.4 | 70 | 8.0 | 42 | 10.4 | 15 | 9.3 |
| [Myocardial](https://docs.wp3a.careme.mtg.pt/definitions/comorbidities.html#myocardial-infarction) infarction | 36 | 8.6 | 780 | 5.1 | 193 | 10.6 | 124 | 14.2 | 64 | 15.9 | 44 | 27.3 |
| [Stroke](https://docs.wp3a.careme.mtg.pt/definitions/comorbidities.html#stroke) | 44 | 10.5 | 1334 | 8.8 | 302 | 16.7 | 185 | 21.2 | 106 | 26.3 | 43 | 26.7 |
| [Ischemic stroke](https://docs.wp3a.careme.mtg.pt/definitions/comorbidities.html#ischemic) | 41 | 9.8 | 1046 | 6.9 | 241 | 13.3 | 153 | 17.5 | 94 | 23.3 | 35 | 21.7 |
| [Hemorrhagic](https://docs.wp3a.careme.mtg.pt/definitions/comorbidities.html#hemorrhagic) stroke | 1 | 0.2 | 146 | 1.0 | 31 | 1.7 | 15 | 1.7 | 15 | 3.7 | 4 | 2.5 |
| [Peripheral artery disease](https://docs.wp3a.careme.mtg.pt/definitions/comorbidities.html#peripheral-artery-disease) | 33 | 7.9 | 819 | 5.4 | 239 | 13.2 | 142 | 16.3 | 79 | 19.6 | 46 | 28.6 |
| [Heart failure](https://docs.wp3a.careme.mtg.pt/definitions/comorbidities.html#stage-latest) | 8 | 1.9 | 410 | 2.7 | 149 | 8.2 | 134 | 15.3 | 101 | 25.1 | 72 | 44.7 |
| [Preserved](https://docs.wp3a.careme.mtg.pt/definitions/comorbidities.html#stage-latest) | 6 | 1.4 | 316 | 2.1 | 113 | 6.2 | 100 | 11.5 | 78 | 19.4 | 54 | 33.5 |
| [Midrange](https://docs.wp3a.careme.mtg.pt/definitions/comorbidities.html#stage-latest) | 2 | 0.5 | 91 | 0.6 | 32 | 1.8 | 28 | 3.2 | 19 | 4.7 | 12 | 7.5 |
| [Reduced](https://docs.wp3a.careme.mtg.pt/definitions/comorbidities.html#stage-latest) | 0 | 0 | 3 | 0 | 4 | 0.2 | 6 | 0.7 | 4 | 1.0 | 6 | 3.7 |
| **Cardiovascular medications** | **n** | **%** | **n** | **%** | **n** | **%** | **n** | **%** | **n** | **%** | **n** | **%** |
| Renin-angiotensin-system-acting agents | 374 | 89.0 | 8698 | 57.1 | 1 551 | 85.5 | 783 | 89.7 | 360 | 89.3 | 139 | 86.3 |
| Angiotensin-converting enzyme inhibitors | 281 | 66.9 | 6177 | 40.6 | 1 147 | 63.3 | 585 | 67.0 | 249 | 61.8 | 105 | 65.2 |
| Angiotensin receptor blockers | 213 | 50.7 | 5106 | 33.5 | 994 | 54.8 | 518 | 59.3 | 268 | 66.5 | 102 | 63.4 |
| Angiotensin receptor-neprilysin inhibitors | 1 | 0.2 | 34 | 0.2 | 21 | 1.2 | 12 | 1.4 | 7 | 1.7 | 0 | 0 |
| Diuretics | 115 | 27.4 | 3619 | 23.8 | 804 | 44.3 | 547 | 62.7 | 306 | 75.9 | 139 | 86.3 |
| Thiazides | 3 | 0.7 | 78 | 0.5 | 16 | 0.9 | 9 | 1.0 | 1 | 0.2 | 0 | 0 |
| Sulfonamides | 47 | 11.2 | 1664 | 10.9 | 493 | 27.2 | 409 | 46.8 | 277 | 68.7 | 134 | 83.2 |
| Aldosterone antagonists | 12 | 2.9 | 438 | 2.9 | 133 | 7.3 | 109 | 12.5 | 63 | 15.6 | 16 | 9.9 |
| Antiplatelets | 158 | 37.6 | 4572 | 30 | 933 | 51.5 | 516 | 59.1 | 254 | 63.0 | 98 | 60.9 |
| Low dose Aspirin® | 149 | 35.5 | 4255 | 27.9 | 862 | 47.5 | 473 | 54.2 | 233 | 57.8 | 93 | 57.8 |
| P2y12 antagonists | 59 | 14.0 | 1459 | 9.6 | 312 | 17.2 | 198 | 22.7 | 100 | 24.8 | 40 | 24.8 |
| Anticoagulants | 31 | 7.4 | 1364 | 9.0 | 348 | 19.2 | 203 | 23.3 | 116 | 28.8 | 47 | 29.2 |
| Vitamin K antagonists | 18 | 4.3 | 712 | 4.7 | 195 | 10.8 | 131 | 15.0 | 87 | 21.6 | 42 | 26.1 |
| Novel oral anticoagulants | 17 | 4.0 | 818 | 5.4 | 201 | 11.1 | 100 | 11.5 | 45 | 11.2 | 8 | 5.0 |
| Calcium channel blockers | 144 | 34.3 | 3128 | 20.5 | 725 | 40 | 488 | 55.9 | 254 | 63.0 | 118 | 73.3 |
| Beta blockers | 136 | 32.4 | 3635 | 23.9 | 747 | 41.2 | 453 | 51.9 | 242 | 60 | 90 | 55.9 |
| Nitrates | 44 | 10.5 | 1014 | 6.7 | 226 | 12.5 | 174 | 19.9 | 109 | 27.0 | 48 | 29.8 |
| **Diabetes medications** | **n** | **%** | **n** | **%** | **n** | **%** | **n** | **%** | **n** | **%** | **n** | **%** |
| Glucose lowering drugs | 374 | 89.0 | 8698 | 57.1 | 1 551 | 85.5 | 783 | 89.7 | 360 | 89.3 | 139 | 86.3 |
| Excluding insulins | 281 | 66.9 | 6177 | 40.6 | 1 147 | 63.3 | 585 | 67.0 | 249 | 61.8 | 105 | 65.2 |
| Biguanides | 213 | 50.7 | 5106 | 33.5 | 994 | 54.8 | 518 | 59.3 | 268 | 66.5 | 102 | 63.4 |
| Sodium-glucose co-transporter-2 inhibitors | 1 | 0.2 | 34 | 0.2 | 21 | 1.2 | 12 | 1.4 | 7 | 1.7 | 0 | 0 |
| Glucagon-like peptide-1 receptor agonists | 115 | 27.4 | 3619 | 23.8 | 804 | 44.3 | 547 | 62.7 | 306 | 75.9 | 139 | 86.3 |
| Dipeptidyl peptidase-4 inhibitors | 3 | 0.7 | 78 | 0.5 | 16 | 0.9 | 9 | 1.0 | 1 | 0.2 | 0 | 0 |
| Glitazones | 47 | 11.2 | 1664 | 10.9 | 493 | 27.2 | 409 | 46.8 | 277 | 68.7 | 134 | 83.2 |
| Sulfonylureas | 12 | 2.9 | 438 | 2.9 | 133 | 7.3 | 109 | 12.5 | 63 | 15.6 | 16 | 9.9 |
| Metiglinides | 158 | 37.6 | 4572 | 30 | 933 | 51.5 | 516 | 59.1 | 254 | 63.0 | 98 | 60.9 |
| Glucosidase inhibitors | 149 | 35.5 | 4255 | 27.9 | 862 | 47.5 | 473 | 54.2 | 233 | 57.8 | 93 | 57.8 |
| Insulins | 59 | 14.0 | 1459 | 9.6 | 312 | 17.2 | 198 | 22.7 | 100 | 24.8 | 40 | 24.8 |
| Long acting | 31 | 7.4 | 1364 | 9.0 | 348 | 19.2 | 203 | 23.3 | 116 | 28.8 | 47 | 29.2 |
| Intermediate acting | 18 | 4.3 | 712 | 4.7 | 195 | 10.8 | 131 | 15.0 | 87 | 21.6 | 42 | 26.1 |
| Fast acting | 17 | 4.0 | 818 | 5.4 | 201 | 11.1 | 100 | 11.5 | 45 | 11.2 | 8 | 5.0 |
| Premixed | 144 | 34.3 | 3128 | 20.5 | 725 | 40 | 488 | 55.9 | 254 | 63.0 | 118 | 73.3 |
| **Bone medications** | **n** | **%** | **n** | **%** | **n** | **%** | **n** | **%** | **n** | **%** | **n** | **%** |
| [Bone Disease Medications](https://docs.wp3a.careme.mtg.pt/definitions/medications.html#bone-disease-medication) | 8 | 1.9 | 480 | 3.2 | 97 | 5.4 | 40 | 4.6 | 14 | 3.5 | 6 | 3.7 |
| [Vitamin D](https://docs.wp3a.careme.mtg.pt/definitions/medications.html#vitamin-d-and-analogues) | 25 | 6.0 | 694 | 4.6 | 156 | 8.6 | 152 | 17.4 | 159 | 39.5 | 56 | 34.8 |
| [Calcium](https://docs.wp3a.careme.mtg.pt/definitions/medications.html#calcium) | 14 | 3.3 | 701 | 4.6 | 127 | 7.0 | 53 | 6.1 | 37 | 9.2 | 27 | 16.8 |
| [Magnesium](https://docs.wp3a.careme.mtg.pt/definitions/medications.html#magnesium) | 46 | 11.0 | 1444 | 9.5 | 248 | 13.7 | 128 | 14.7 | 60 | 14.9 | 27 | 16.8 |
| [Estrogens and progestogens](https://docs.wp3a.careme.mtg.pt/definitions/medications.html#estrogens-and-progestogens) | 0 | 0 | 0 | 0 | 0 | 0 | 0 | 0 | 0 | 0 | 0 | 0 |
| [Calcitonins](https://docs.wp3a.careme.mtg.pt/definitions/medications.html#calcitonins) | 1 | 0.2 | 25 | 0.2 | 4 | 0.2 | 0 | 0 | 2 | 0.5 | 1 | 0.6 |

**Table S3** - Detailed characterization of CKD female population according to KDIGO guidelines using UACR (mg/g). In total, 16379 (21.0%) patients had at least two UARC assessments. Percentages are presented for the overall female population (n=77126).

| **n=16379 (21.0%)** | **UACR < 30**  **14537 (18.9%)** | | **UACR 30-300**  **1403 (1.8%)** | | **UACR ≥ 300**  **439 (0.6%)** | |
| --- | --- | --- | --- | --- | --- | --- |
| **Sociodemographic characteristics** | **n** | **%** | **n** | **%** | **n** | **%** |
| [Age](https://docs.wp3a.careme.mtg.pt/definitions/observations.html#age) (years) | 68.0 (P50) | 17.0 (IRQ) | 75.0 (P50) | 19.0 (IRQ) | 74.0 (P50) | 18.0 (IRQ) |
| [[20, 79]](https://docs.wp3a.careme.mtg.pt/definitions/observations.html#between-20-and-79) | 11804 | 81.2 | 832 | 59.3 | 288 | 65.6 |
| [Between 50 and 60](https://docs.wp3a.careme.mtg.pt/definitions/observations.html#between-50-and-60) | 2484 | 17.1 | 152 | 10.8 | 48 | 10.9 |
| [Between 60 and 70](https://docs.wp3a.careme.mtg.pt/definitions/observations.html#between-60-and-70) | 4227 | 29.1 | 265 | 18.9 | 85 | 19.4 |
| [Between 70 and 80](https://docs.wp3a.careme.mtg.pt/definitions/observations.html#between-70-and-80) | 4005 | 27.6 | 341 | 24.3 | 124 | 28.3 |
| [Aged 80 or](https://docs.wp3a.careme.mtg.pt/definitions/observations.html#aged-80-and-over) more | 2722 | 18.7 | 569 | 40.6 | 150 | 34.2 |
| **Body mass index (Kg/m^2^)** | **n** | **%** | **n** | **%** | **n** | **%** |
| [BMI < 18.5](https://docs.wp3a.careme.mtg.pt/definitions/observations.html#less-than-185) | 120 | 0.8 | 28 | 2,0 | 7 | 1,6 |
| [BMI [18.5, 25.0[](https://docs.wp3a.careme.mtg.pt/definitions/observations.html#between-185-and-250) | 2888 | 19.9 | 290 | 20.7 | 101 | 23.0 |
| [BMI [25.0, 30.0[](https://docs.wp3a.careme.mtg.pt/definitions/observations.html#between-250-and-300) | 5689 | 39.1 | 497 | 35.4 | 149 | 33.9 |
| [BMI [30.0, 35.0[](https://docs.wp3a.careme.mtg.pt/definitions/observations.html#between-300-and-350) | 3844 | 26.4 | 350 | 24.9 | 91 | 20.7 |
| [BMI ≥ 20](https://docs.wp3a.careme.mtg.pt/definitions/observations.html#greater-than-350) | 1882 | 12.9 | 214 | 15.3 | 81 | 18.5 |
| **Clinical measurements** | **P50** | **IQR** | **P50** | **IQR** | **P50** | **IQR** |
| Weight (Kg) | 70 | 18.0 | 70 | 20 | 69.0 | 20 |
| BMI (Kg/m^2^) | 28.6 | 6.5 | 28.8 | 7.2 | 28.9 | 8.3 |
| Waist circumference (cm) | 99.0 | 15.0 | 102.0 | 17.0 | 103.0 | 15.0 |
| Systolic blood pressure (mmHg) | 135.0 | 17.0 | 137.0 | 21.0 | 142.0 | 21.0 |
| Diastolic blood pressure (mmHg) | 80 | 12.0 | 78.0 | 15.0 | 79.0 | 15.0 |
| **Laboratory measurements** | **P50** | **IQR** | **P50** | **IQR** | **P50** | **IQR** |
| [Hemoglobin (g/dL](https://docs.wp3a.careme.mtg.pt/definitions/measurements.html#hemoglobin)) | 13.9 | 16.8 | 13.2 | 13.1 | 12.3 | 3.4 |
| [Sodium (mEq/L](https://docs.wp3a.careme.mtg.pt/definitions/measurements.html#sodium)) | 140 | 3.0 | 140 | 4.0 | 140 | 4.0 |
| [Potassium (mEq/L](https://docs.wp3a.careme.mtg.pt/definitions/measurements.html#potassium) | 4.3 | 0.5 | 4.4 | 0.6 | 4.5 | 0.8 |
| [Phosphate (mg/dL](https://docs.wp3a.careme.mtg.pt/definitions/measurements.html#phosphate)) | 3.4 | 0.7 | 3.4 | 0.9 | 3.7 | 1.1 |
| [Magnesium (mg/dL](https://docs.wp3a.careme.mtg.pt/definitions/measurements.html#magnesium)) | 2.1 | 0.3 | 2.0 | 0.4 | 2.0 | 0.4 |
| [Calcium (mg/dL](https://docs.wp3a.careme.mtg.pt/definitions/measurements.html#calcium)) | 9.4 | 0.6 | 9.4 | 0.8 | 9.4 | 1.0 |
| [Vitamin D (ng/mL](https://docs.wp3a.careme.mtg.pt/definitions/measurements.html#vitamin-d) | 19.0 | 14.0 | 16.0 | 17.0 | 18.0 | 15.5 |
| [Uric acid (µg/dL](https://docs.wp3a.careme.mtg.pt/definitions/measurements.html#uric-acid)) | 4.8 | 1.8 | 5.6 | 2.5 | 6.3 | 2.7 |
| [Creatine kinase (IU/L](https://docs.wp3a.careme.mtg.pt/definitions/measurements.html#creatine-kinase)) | 78.0 | 59.0 | 72.0 | 65.0 | 69.0 | 63.0 |
| [Iron (µg/L](https://docs.wp3a.careme.mtg.pt/definitions/measurements.html#total-serum-iron)) | 76.0 | 41.0 | 61.0 | 38.0 | 58.0 | 37.0 |
| [Transferrin (µg/L](https://docs.wp3a.careme.mtg.pt/definitions/measurements.html#transferrin)) | 258.0 | 79.0 | 251.0 | 93.0 | 224.0 | 90 |
| [Total iron binding capacity (µg/L](https://docs.wp3a.careme.mtg.pt/definitions/measurements.html#total-iron-binding-capacity)) | 307.0 | 87.0 | 289.0 | 103.0 | 271.0 | 83.0 |
| [Parathyroid hormone (pg/mL](https://docs.wp3a.careme.mtg.pt/definitions/measurements.html#parathyroid-hormone)) | 59.1 | 48.4 | 97.3 | 133.5 | 149.5 | 140.4 |
| [Glucose (mg/dL](https://docs.wp3a.careme.mtg.pt/definitions/measurements.html#glucose)) | 103.0 | 37.4 | 123.0 | 64.8 | 134.1 | 85.8 |
| [HbA1c (%](https://docs.wp3a.careme.mtg.pt/definitions/measurements.html#glycated-hemoglobin)) | 6.5 | 30.3 | 7.5 | 33.9 | 8.0 | 32.8 |
| [LDL-cholesterol (mg/dL)](https://docs.wp3a.careme.mtg.pt/definitions/measurements.html#cholesterol-ldl) | 117.0 | 43.0 | 105.0 | 45.0 | 103.0 | 49.0 |
| [HDL-cholesterol (mg/dL)](https://docs.wp3a.careme.mtg.pt/definitions/measurements.html#cholesterol-hdl) | 49.0 | 16.0 | 45.0 | 15.0 | 42.0 | 19.0 |
| [Non-HDL-cholesterol (mg/dL](https://docs.wp3a.careme.mtg.pt/definitions/measurements.html#cholesterol-non-hdl)) | 138.0 | 45.0 | 129.0 | 49.0 | 134.0 | 55.5 |
| [Total cholesterol (mg/dL](https://docs.wp3a.careme.mtg.pt/definitions/measurements.html#cholesterol-total)) | 190 | 46.0 | 176.0 | 51.0 | 179.0 | 60 |
| [Triglycerides (mg/dL)](https://docs.wp3a.careme.mtg.pt/definitions/measurements.html#triglycerides) | 107.0 | 61.0 | 122.0 | 75.0 | 135.0 | 93.0 |
| Brain natriuretic peptide (pg/mL) | 96.0 | 180.1 | 174.3 | 355.0 | 185.7 | 355.8 |
| NT-pro-brain natriuretic peptide (pg/mL) | 1348.5 | 4049.7 | 1679.5 | 3184.2 | 3056.5 | 7653.0 |
| [Albumin (g/dL](https://docs.wp3a.careme.mtg.pt/definitions/measurements.html#albumin)) | 4.4 | 41.1 | 4.2 | 1.0 | 4.0 | 1.0 |
| [Bilirubin (mg/dL](https://docs.wp3a.careme.mtg.pt/definitions/measurements.html#bilirubin)) | 0.6 | 0.3 | 0.5 | 0.3 | 0.5 | 0.3 |
| [ALT (IU/L](https://docs.wp3a.careme.mtg.pt/definitions/measurements.html#alanine-amino-transferase)) | 18.0 | 10 | 17.0 | 11.0 | 17.0 | 10 |
| [AST (IU/L](https://docs.wp3a.careme.mtg.pt/definitions/measurements.html#aspartate-amino-transferase)) | 19.0 | 7.0 | 19.0 | 8.0 | 19.0 | 10 |
| [Alkaline phosphatase (IU/L](https://docs.wp3a.careme.mtg.pt/definitions/measurements.html#alkaline-phosphatase)) | 74.0 | 32.0 | 81.0 | 36.0 | 89.0 | 43.0 |
| [TSH (IU/mL](https://docs.wp3a.careme.mtg.pt/definitions/measurements.html#thyroid-stimulating-hormone)) | 1.7 | 1.3 | 1.7 | 1.4 | 1.7 | 1.6 |
| [T3 (µg/dL](https://docs.wp3a.careme.mtg.pt/definitions/measurements.html#triiodothyronine)) | 2.8 | 0.7 | 2.7 | 0.7 | 2.6 | 0.9 |
| [T4 (µg/dL](https://docs.wp3a.careme.mtg.pt/definitions/measurements.html#thyroxine)) | 1.0 | 0.2 | 1.0 | 0.2 | 1.0 | 0.2 |
| [Creatinine (mg/dL](https://docs.wp3a.careme.mtg.pt/definitions/measurements.html#creatinine)) | 0.8 | 0.1 | 0.9 | 0.5 | 1.2 | 1.3 |
| **Comorbidities** | **n** | **%** | **n** | **%** | **n** | **%** |
| [Obesity](https://docs.wp3a.careme.mtg.pt/definitions/comorbidities.html#obesity) | 5730 | 39.3 | 564 | 40.1 | 172 | 39.2 |
| [Hypercholesterolemia](https://docs.wp3a.careme.mtg.pt/definitions/comorbidities.html#hypercholesterolemia) | 7224 | 49.5 | 493 | 35.1 | 178 | 40.5 |
| [Type 2 diabetes](https://docs.wp3a.careme.mtg.pt/definitions/comorbidities.html#diabetes-mellitus-type-2) mellitus | 6874 | 47.1 | 1042 | 74.2 | 372 | 84.7 |
| [Structural heart disease](https://docs.wp3a.careme.mtg.pt/definitions/comorbidities.html#heart-disease-structural) | 3043 | 20.9 | 570 | 40.6 | 238 | 54.2 |
| [Microvascular disease](https://docs.wp3a.careme.mtg.pt/definitions/comorbidities.html#microvascular-disease) | 1276 | 8.7 | 287 | 20.4 | 177 | 40.3 |
| [Cardiovascular disease](https://docs.wp3a.careme.mtg.pt/definitions/comorbidities.html#cardiovascular-disease) | 13905 | 95.3 | 1379 | 98.1 | 432 | 98.4 |
| [Hypertension](https://docs.wp3a.careme.mtg.pt/definitions/comorbidities.html#hypertension) | 13733 | 94.2 | 1344 | 95.7 | 421 | 95.9 |
| [Atrial fibrillation](https://docs.wp3a.careme.mtg.pt/definitions/comorbidities.html#atrial-fibrillation) | 1016 | 7.0 | 267 | 19.0 | 85 | 19.4 |
| [Chronic](https://docs.wp3a.careme.mtg.pt/definitions/comorbidities.html#chronic-kidney-disease) kidney disease | 2088 | 14.3 | 598 | 42.6 | 290 | 66.1 |
| [Stable angina](https://docs.wp3a.careme.mtg.pt/definitions/comorbidities.html#angina-stable) | 514 | 3.5 | 116 | 8.3 | 51 | 11.6 |
| [Transient ischemic attack](https://docs.wp3a.careme.mtg.pt/definitions/comorbidities.html#transient-ischemic-attack) | 167 | 1.1 | 40 | 2.8 | 17 | 3.9 |
| [Arterosclerotic disease](https://docs.wp3a.careme.mtg.pt/definitions/comorbidities.html#arterosclerotic-disease) | 1901 | 13.0 | 374 | 26.6 | 155 | 35.3 |
| [Unstable angina](https://docs.wp3a.careme.mtg.pt/definitions/comorbidities.html#angina-unstable) | 282 | 1.9 | 46 | 3.3 | 22 | 5.0 |
| [Miocardial](https://docs.wp3a.careme.mtg.pt/definitions/comorbidities.html#myocardial-infarction) infarction | 341 | 2.3 | 86 | 6.1 | 45 | 10.3 |
| [Stroke](https://docs.wp3a.careme.mtg.pt/definitions/comorbidities.html#stroke) | 1326 | 9.1 | 264 | 18.8 | 122 | 27.8 |
| [Ischemic stroke](https://docs.wp3a.careme.mtg.pt/definitions/comorbidities.html#ischemic) | 1093 | 7.5 | 220 | 15.7 | 105 | 23.9 |
| [Hemorrhagic](https://docs.wp3a.careme.mtg.pt/definitions/comorbidities.html#hemorrhagic) stroke | 92 | 0.6 | 27 | 1.9 | 5 | 1.1 |
| [Peripheral artery disease](https://docs.wp3a.careme.mtg.pt/definitions/comorbidities.html#peripheral-artery-disease) | 526 | 3.6 | 111 | 7.9 | 43 | 9.8 |
| [Heart failure](https://docs.wp3a.careme.mtg.pt/definitions/comorbidities.html#stage-latest) | 509 | 3.5 | 169 | 12.0 | 97 | 22.1 |
| [Preserved](https://docs.wp3a.careme.mtg.pt/definitions/comorbidities.html#stage-latest) | 442 | 3.0 | 141 | 10 | 87 | 19.8 |
| [Midrange](https://docs.wp3a.careme.mtg.pt/definitions/comorbidities.html#stage-latest) | 61 | 0.4 | 26 | 1.9 | 10 | 2.3 |
| [Reduced](https://docs.wp3a.careme.mtg.pt/definitions/comorbidities.html#stage-latest) | 6 | 0 | 2 | 0.1 | 0 | 0 |
| **Cardiovascular medications** | **n** | **%** | **n** | **%** | **n** | **%** |
| Renin-angiotensin-system-acting agents | 12548 | 86.0 | 1284 | 91.4 | 413 | 94.1 |
| Angiotensin-converting enzyme inhibitors | 8656 | 59.4 | 973 | 69.3 | 335 | 76.3 |
| Angiotensin receptor blockers | 8297 | 56.9 | 897 | 63.8 | 312 | 71.1 |
| Angiotensin receptor-neprilysin inhibitors | 14 | 0.1 | 4 | 0.3 | 3 | 0.7 |
| Diuretics | 7020 | 48.1 | 907 | 64.6 | 335 | 76.3 |
| Thiazydes | 126 | 0.9 | 9 | 0.6 | 5 | 1.1 |
| Sulfonamides | 3429 | 23.5 | 656 | 46.7 | 270 | 61.5 |
| Aldosterone antagonists | 646 | 4.4 | 127 | 9.0 | 54 | 12.3 |
| Antiplatelets | 4228 | 29.0 | 678 | 48.3 | 256 | 58.3 |
| Low dose Aspirin® | 3861 | 26.5 | 626 | 44.6 | 240 | 54.7 |
| P2y12 antagonists | 986 | 6.8 | 191 | 13.6 | 80 | 18.2 |
| Anticoagulants | 1310 | 9.0 | 279 | 19.9 | 80 | 18.2 |
| Vitamin K antagonists | 671 | 4.6 | 202 | 14.4 | 62 | 14.1 |
| Novel oral anticoagulants | 807 | 5.5 | 132 | 9.4 | 35 | 8.0 |
| Calcium channel blockers | 4633 | 31.8 | 770 | 54.8 | 307 | 69.9 |
| Beta blockers | 5230 | 35.9 | 671 | 47.8 | 242 | 55.1 |
| Nitrates | 812 | 5.6 | 157 | 11.2 | 70 | 15.9 |
| **Diabetes medications** | **n** | **%** | **n** | **%** | **n** | **%** |
| Glucose lowering drugs | 5103 | 35.0 | 855 | 60.9 | 316 | 72.0 |
| Excluding insulins | 5032 | 34.5 | 836 | 59.5 | 308 | 70.2 |
| Biguanides | 4801 | 32.9 | 799 | 56.9 | 285 | 64.9 |
| Sodium-glucose co-transporter-2 inhibitors | 542 | 3.7 | 122 | 8.7 | 28 | 6.4 |
| Glucagon-like peptide-1 receptor agonists | 252 | 1.7 | 55 | 3.9 | 26 | 5.9 |
| Dipeptidyl peptidase-4 inhibitors | 2100 | 14.4 | 520 | 37.0 | 232 | 52.8 |
| Glitazones | 398 | 2.7 | 126 | 9.0 | 88 | 20 |
| Sulfonylureas | 1823 | 12.5 | 459 | 32.7 | 200 | 45.6 |
| Metiglinides | 64 | 0.4 | 19 | 1.4 | 12 | 2.7 |
| Glucosidase inhibitors | 315 | 2.2 | 124 | 8.8 | 56 | 12.8 |
| Insulins | 752 | 5.2 | 309 | 22.0 | 184 | 41.9 |
| Long acting | 540 | 3.7 | 212 | 15.1 | 130 | 29.6 |
| Intermediate acting | 328 | 2.2 | 147 | 10.5 | 87 | 19.8 |
| Fast acting | 230 | 1.6 | 77 | 5.5 | 63 | 14.4 |
| Premixed | 234 | 1.6 | 82 | 5.8 | 60 | 13.7 |
| **Bone medications** | **n** | **%** | **n** | **%** | **n** | **%** |
| [Bone Disease Medications](https://docs.wp3a.careme.mtg.pt/definitions/medications.html#bone-disease-medication) | 3987 | 27.3 | 373 | 26.5 | 91 | 20.7 |
| [Vitamin D](https://docs.wp3a.careme.mtg.pt/definitions/medications.html#vitamin-d-and-analogues) | 2642 | 18.1 | 331 | 23.6 | 145 | 33.0 |
| [Calcium](https://docs.wp3a.careme.mtg.pt/definitions/medications.html#calcium) | 3956 | 27.1 | 391 | 27.8 | 114 | 26.0 |
| [Magnesium](https://docs.wp3a.careme.mtg.pt/definitions/medications.html#magnesium) | 3068 | 21.0 | 282 | 20.1 | 92 | 21.0 |
| [Estrogens and progestogens](https://docs.wp3a.careme.mtg.pt/definitions/medications.html#estrogens-and-progestogens) | 5995 | 41.1 | 456 | 32.5 | 139 | 31.7 |
| [Calcitonins](https://docs.wp3a.careme.mtg.pt/definitions/medications.html#calcitonins) | 194 | 1.3 | 16 | 1.1 | 4 | 0.9 |

**Table S4** - Detailed characterization of CKD male population according to KDIGO guidelines using UACR (mg/g). In total, 14155 (23.6%) patients had at least two UARC assessments. Percentages are presented for the overall male population (n=59867).

| **n=59867 (23.6%)** | **UACR < 30**  **11961 (20.0%)** | | **UACR 30-300**  **1529 (2.6%)** | | **UACR ≥ 300**  **665 (1.1%)** | |
| --- | --- | --- | --- | --- | --- | --- |
| **Sociodemographic characteristics** | **n** | **%** | **n** | **%** | **n** | **%** |
| [Age](https://docs.wp3a.careme.mtg.pt/definitions/observations.html#age) (years) | 67.0 (P50) | 16.0 (IRQ) | 71.0 (P50) | 18.0 (IRQ) | 71.0 (P50) | 18.0 (IRQ) |
| [[20, 79]](https://docs.wp3a.careme.mtg.pt/definitions/observations.html#between-20-and-79) | 10348 | 86.5 | 1159 | 75.8 | 496 | 74.6 |
| [Between 50 and 60](https://docs.wp3a.careme.mtg.pt/definitions/observations.html#between-50-and-60) | 2245 | 18.8 | 224 | 14,7 | 80 | 12.0 |
| [Between 60 and 70](https://docs.wp3a.careme.mtg.pt/definitions/observations.html#between-60-and-70) | 3687 | 30.8 | 390 | 25.5 | 178 | 26.8 |
| [Between 70 and 80](https://docs.wp3a.careme.mtg.pt/definitions/observations.html#between-70-and-80) | 3288 | 27.5 | 454 | 29.7 | 199 | 29.9 |
| [Aged 80 or](https://docs.wp3a.careme.mtg.pt/definitions/observations.html#aged-80-and-over) more | 1600 | 13.4 | 370 | 24.2 | 169 | 25.4 |
| **Body mass index (Kg/m^2^)** | **n** | **%** | **n** | **%** | **n** | **%** |
| [BMI < 18.5](https://docs.wp3a.careme.mtg.pt/definitions/observations.html#less-than-185) | 91 | 0.8 | 11 | 0.7 | 9 | 1.4 |
| [BMI [18.5, 25.0[](https://docs.wp3a.careme.mtg.pt/definitions/observations.html#between-185-and-250) | 2851 | 23.8 | 340 | 22.2 | 144 | 21.7 |
| [BMI [25.0, 30.0[](https://docs.wp3a.careme.mtg.pt/definitions/observations.html#between-250-and-300) | 5588 | 46.7 | 694 | 45.4 | 287 | 43.2 |
| [BMI [30.0, 35.0[](https://docs.wp3a.careme.mtg.pt/definitions/observations.html#between-300-and-350) | 2608 | 21.8 | 353 | 23.1 | 147 | 22.1 |
| [BMI ≥ 20](https://docs.wp3a.careme.mtg.pt/definitions/observations.html#greater-than-350) | 694 | 5.8 | 111 | 7.3 | 60 | 9.0 |
| **Clinical measurements** | **P50** | **IQR** | **P50** | **IQR** | **P50** | **IQR** |
| Weight (Kg) | 110 | 0.9 | 12 | 0.8 | 10 | 1.5 |
| BMI (Kg/m^2^) | 2860 | 23.8 | 340 | 22.2 | 144 | 21.6 |
| Waist circumference (cm) | 5591 | 46.6 | 694 | 45.3 | 287 | 43.1 |
| Systolic blood pressure (mmHg) | 2610 | 21.8 | 353 | 23.0 | 147 | 22.1 |
| Diastolic blood pressure (mmHg) | 695 | 5.8 | 112 | 7.3 | 60 | 9.0 |
| **Echocardiography measurements** | **P50** | **IQR** | **P50** | **IQR** | **P50** | **IQR** |
| Left atrial volume (mL) | 40 | 7.0 | 42.0 | 7.0 | 42.0 | 7.0 |
| Left atrial volume index (mL/m^2^) | 21.2 | 3.8 | 22.3 | 4.8 | 22.0 | 4.3 |
| Left ventricular mass (g) | 145.3 | 40.2 | 154.3 | 43.9 | 162.9 | 51.1 |
| Left ventricular mass index (g/m^2^) | 77.3 | 21.2 | 81.7 | 23.2 | 85.9 | 27.0 |
| Left atrial diameter (mm) | 51.0 | 6.0 | 51.0 | 7.0 | 52.0 | 6.0 |
| Ejection fraction (%) | 61.0 | 8.0 | 60 | 9.0 | 60 | 9.0 |
| Left ventricular posterior wall thickness (mL) | 9.0 | 1.0 | 10 | 1.0 | 10 | 2.0 |
| Interventricular septum thickness (mm) | 10 | 1.0 | 11.0 | 2.0 | 11.0 | 3.0 |
| **Laboratory measurements** | **P50** | **IQR** | **P50** | **IQR** | **P50** | **IQR** |
| [Hemoglobin (g/dL](https://docs.wp3a.careme.mtg.pt/definitions/measurements.html#hemoglobin)) | 15.3 | 16.3 | 14.7 | 12.5 | 13.8 | 4.0 |
| [Sodium (mEq/L](https://docs.wp3a.careme.mtg.pt/definitions/measurements.html#sodium)) | 140 | 3.0 | 140 | 3.0 | 140 | 4.0 |
| [Potassium (mEq/L](https://docs.wp3a.careme.mtg.pt/definitions/measurements.html#potassium) | 4.3 | 0.5 | 4.4 | 0.6 | 4.5 | 0.8 |
| [Phosphate (mg/dL](https://docs.wp3a.careme.mtg.pt/definitions/measurements.html#phosphate)) | 3.2 | 0.8 | 3.3 | 0.8 | 3.5 | 1.0 |
| [Magnesium (mg/dL](https://docs.wp3a.careme.mtg.pt/definitions/measurements.html#magnesium)) | 2.1 | 0.3 | 2.0 | 0.3 | 2.0 | 0.4 |
| [Calcium (mg/dL](https://docs.wp3a.careme.mtg.pt/definitions/measurements.html#calcium)) | 9.4 | 0.6 | 9.5 | 0.7 | 9.3 | 0.9 |
| [Vitamin D (ng/mL](https://docs.wp3a.careme.mtg.pt/definitions/measurements.html#vitamin-d) | 19.0 | 15.0 | 18.0 | 14.0 | 18.0 | 16.0 |
| [Uric acid (µg/dL](https://docs.wp3a.careme.mtg.pt/definitions/measurements.html#uric-acid)) | 5.9 | 1.9 | 6.3 | 2.3 | 6.7 | 2.5 |
| [Creatine kinase (IU/L](https://docs.wp3a.careme.mtg.pt/definitions/measurements.html#creatine-kinase)) | 95.0 | 87.0 | 86.5 | 83.8 | 91.0 | 100 |
| [Iron (µg/L](https://docs.wp3a.careme.mtg.pt/definitions/measurements.html#total-serum-iron)) | 87.0 | 49.0 | 70 | 48.5 | 69.0 | 43.5 |
| [Transferrin (µg/L](https://docs.wp3a.careme.mtg.pt/definitions/measurements.html#transferrin)) | 242.0 | 74.3 | 234.0 | 71.5 | 222.5 | 74.3 |
| [Total iron binding capacity (µg/L](https://docs.wp3a.careme.mtg.pt/definitions/measurements.html#total-iron-binding-capacity)) | 292.0 | 78.0 | 285.5 | 85.5 | 264.0 | 74.0 |
| [Parathyroid hormone (pg/mL](https://docs.wp3a.careme.mtg.pt/definitions/measurements.html#parathyroid-hormone)) | 54.1 | 51.4 | 80.1 | 74.4 | 114.8 | 122.1 |
| [Glucose (mg/dL](https://docs.wp3a.careme.mtg.pt/definitions/measurements.html#glucose)) | 109.0 | 39.3 | 129.0 | 59.9 | 130 | 65.6 |
| [HbA1c (%](https://docs.wp3a.careme.mtg.pt/definitions/measurements.html#glycated-hemoglobin)) | 6.6 | 30.3 | 7.5 | 33.9 | 8.1 | 33.9 |
| [LDL-cholesterol (mg/dL)](https://docs.wp3a.careme.mtg.pt/definitions/measurements.html#cholesterol-ldl) | 109.0 | 43.4 | 99.0 | 46.0 | 97.0 | 49.0 |
| [HDL-cholesterol (mg/dL)](https://docs.wp3a.careme.mtg.pt/definitions/measurements.html#cholesterol-hdl) | 43.0 | 15.0 | 41.0 | 15.0 | 40 | 17.0 |
| [Non-HDL-cholesterol (mg/dL](https://docs.wp3a.careme.mtg.pt/definitions/measurements.html#cholesterol-non-hdl)) | 131.0 | 47.0 | 124.0 | 51.0 | 124.0 | 52.8 |
| [Total cholesterol (mg/dL](https://docs.wp3a.careme.mtg.pt/definitions/measurements.html#cholesterol-total)) | 177.0 | 49.0 | 167.0 | 54.0 | 168.0 | 56.8 |
| [Triglycerides (mg/dL)](https://docs.wp3a.careme.mtg.pt/definitions/measurements.html#triglycerides) | 111.0 | 70 | 120 | 76.0 | 130.5 | 91.8 |
| Brain natriuretic peptide (pg/mL) | 86.7 | 156.8 | 157.1 | 328.4 | 167.9 | 408.7 |
| NT-pro-brain natriuretic peptide (pg/mL) | 755.3 | 1538.0 | 1110.2 | 4659.3 | 4676.8 | 6082.7 |
| [Albumin (g/dL](https://docs.wp3a.careme.mtg.pt/definitions/measurements.html#albumin)) | 4.5 | 25.4 | 4.3 | 0.9 | 4.1 | 0.9 |
| [Bilirubin (mg/dL](https://docs.wp3a.careme.mtg.pt/definitions/measurements.html#bilirubin)) | 0.7 | 0.5 | 0.6 | 0.4 | 0.6 | 0.4 |
| [ALT (IU/L](https://docs.wp3a.careme.mtg.pt/definitions/measurements.html#alanine-amino-transferase)) | 23.0 | 15.0 | 21.0 | 15.0 | 21.0 | 17.0 |
| [AST (IU/L](https://docs.wp3a.careme.mtg.pt/definitions/measurements.html#aspartate-amino-transferase)) | 21.0 | 9.0 | 21.0 | 10 | 20 | 11.0 |
| [Alkaline phosphatase (IU/L](https://docs.wp3a.careme.mtg.pt/definitions/measurements.html#alkaline-phosphatase)) | 67.0 | 28.0 | 73.5 | 33.3 | 80 | 42.0 |
| [TSH (IU/mL](https://docs.wp3a.careme.mtg.pt/definitions/measurements.html#thyroid-stimulating-hormone)) | 1.5 | 1.1 | 1.5 | 1.1 | 1.6 | 1.3 |
| [T3 (µg/dL](https://docs.wp3a.careme.mtg.pt/definitions/measurements.html#triiodothyronine)) | 2.9 | 0.7 | 2.8 | 0.8 | 2.7 | 0.8 |
| [T4 (µg/dL](https://docs.wp3a.careme.mtg.pt/definitions/measurements.html#thyroxine)) | 1.0 | 0.2 | 1.0 | 0.2 | 1.0 | 0.2 |
| [Creatinine (mg/dL](https://docs.wp3a.careme.mtg.pt/definitions/measurements.html#creatinine)) | 0.9 | 0.2 | 1.0 | 0.5 | 1.4 | 1.3 |
| **Comorbidities** | **n** | **%** | **n** | **%** | **n** | **%** |
| [Obesity](https://docs.wp3a.careme.mtg.pt/definitions/comorbidities.html#obesity) | 3305 | 27.5 | 465 | 30.4 | 207 | 31.1 |
| [Hypercholesterolemia](https://docs.wp3a.careme.mtg.pt/definitions/comorbidities.html#hypercholesterolemia) | 4287 | 35.7 | 442 | 28.9 | 209 | 31.4 |
| [Type 2 diabetes](https://docs.wp3a.careme.mtg.pt/definitions/comorbidities.html#diabetes-mellitus-type-2) mellitus | 6395 | 53.3 | 1174 | 76.6 | 561 | 84.2 |
| [Structural heart disease](https://docs.wp3a.careme.mtg.pt/definitions/comorbidities.html#heart-disease-structural) | 2872 | 23.9 | 616 | 40.2 | 377 | 56.6 |
| [Microvascular disease](https://docs.wp3a.careme.mtg.pt/definitions/comorbidities.html#microvascular-disease) | 527 | 4.4 | 275 | 18.0 | 199 | 29.9 |
| [Cardiovascular disease](https://docs.wp3a.careme.mtg.pt/definitions/comorbidities.html#cardiovascular-disease) | 11358 | 94.7 | 1493 | 97.5 | 659 | 98.9 |
| [Hypertension](https://docs.wp3a.careme.mtg.pt/definitions/comorbidities.html#hypertension) | 11140 | 92.9 | 1456 | 95.0 | 635 | 95.3 |
| [Atrial fibrillation](https://docs.wp3a.careme.mtg.pt/definitions/comorbidities.html#atrial-fibrillation) | 882 | 7.4 | 238 | 15.5 | 117 | 17.6 |
| [Chronic](https://docs.wp3a.careme.mtg.pt/definitions/comorbidities.html#chronic-kidney-disease) kidney disease | 1363 | 11.4 | 495 | 32.3 | 393 | 59.0 |
| [Stable angina](https://docs.wp3a.careme.mtg.pt/definitions/comorbidities.html#angina-stable) | 1063 | 8.9 | 258 | 16.8 | 135 | 20.3 |
| [Transient ischemic attack](https://docs.wp3a.careme.mtg.pt/definitions/comorbidities.html#transient-ischemic-attack) | 185 | 1.5 | 42 | 2.7 | 19 | 2.9 |
| [Arterosclerotic disease](https://docs.wp3a.careme.mtg.pt/definitions/comorbidities.html#arterosclerotic-disease) | 2367 | 19.7 | 549 | 35.8 | 277 | 41.6 |
| [Unstable angina](https://docs.wp3a.careme.mtg.pt/definitions/comorbidities.html#angina-unstable) | 513 | 4.3 | 133 | 8.7 | 53 | 8.0 |
| [Miocardial](https://docs.wp3a.careme.mtg.pt/definitions/comorbidities.html#myocardial-infarction) infarction | 824 | 6.9 | 178 | 11.6 | 89 | 13.4 |
| [Stroke](https://docs.wp3a.careme.mtg.pt/definitions/comorbidities.html#stroke) | 1147 | 9.6 | 280 | 18.3 | 147 | 22.1 |
| [Ischemic stroke](https://docs.wp3a.careme.mtg.pt/definitions/comorbidities.html#ischemic) | 888 | 7.4 | 242 | 15.8 | 123 | 18.5 |
| [Hemorrhagic](https://docs.wp3a.careme.mtg.pt/definitions/comorbidities.html#hemorrhagic) stroke | 120 | 1.0 | 29 | 1.9 | 17 | 2.6 |
| [Peripheral artery disease](https://docs.wp3a.careme.mtg.pt/definitions/comorbidities.html#peripheral-artery-disease) | 823 | 6.9 | 221 | 14.4 | 142 | 21.3 |
| [Heart failure](https://docs.wp3a.careme.mtg.pt/definitions/comorbidities.html#stage-latest) | 391 | 3.3 | 159 | 10.4 | 129 | 19.4 |
| [Preserved](https://docs.wp3a.careme.mtg.pt/definitions/comorbidities.html#stage-latest) | 302 | 2.5 | 121 | 7.9 | 103 | 15.5 |
| [Midrange](https://docs.wp3a.careme.mtg.pt/definitions/comorbidities.html#stage-latest) | 78 | 0.7 | 36 | 2.3 | 22 | 3.3 |
| [Reduced](https://docs.wp3a.careme.mtg.pt/definitions/comorbidities.html#stage-latest) | 11 | 0.1 | 2 | 0.1 | 4 | 0.6 |
| **Cardiovascular medications** | **n** | **%** | **n** | **%** | **n** | **%** |
| Renin-angiotensin-system-acting agents | 10221 | 85.2 | 1423 | 92.9 | 636 | 95.5 |
| Angiotensin-converting enzyme inhibitors | 7329 | 61.1 | 1072 | 70 | 492 | 73.9 |
| Angiotensin receptor blockers | 6018 | 50.2 | 914 | 59.7 | 460 | 69.1 |
| Angiotensin receptor-neprilysin inhibitors | 39 | 0.3 | 8 | 0.5 | 2 | 0.3 |
| Diuretics | 3843 | 32.0 | 681 | 44.5 | 419 | 62.9 |
| Thiazydes | 88 | 0.7 | 12 | 0.8 | 8 | 1.2 |
| Sulfonamides | 1597 | 13.3 | 426 | 27.8 | 321 | 48.2 |
| Aldosterone antagonists | 445 | 3.7 | 89 | 5.8 | 68 | 10.2 |
| Antiplatelets | 4441 | 37.0 | 840 | 54.8 | 417 | 62.6 |
| Low dose Aspirin® | 4141 | 34.5 | 792 | 51.7 | 389 | 58.4 |
| P2y12 antagonists | 1463 | 12.2 | 289 | 18.9 | 160 | 24.0 |
| Anticoagulants | 1075 | 9.0 | 270 | 17.6 | 143 | 21.5 |
| Vitamin K antagonists | 565 | 4.7 | 177 | 11.6 | 96 | 14.4 |
| Novel oral anticoagulants | 644 | 5.4 | 141 | 9.2 | 64 | 9.6 |
| Calcium channel blockers | 3493 | 29.1 | 764 | 49.9 | 450 | 67.6 |
| Beta blockers | 3753 | 31.3 | 667 | 43.5 | 348 | 52.3 |
| Nitrates | 1017 | 8.5 | 245 | 16.0 | 123 | 18.5 |
| **Diabetes medications** | **n** | **%** | **n** | **%** | **n** | **%** |
| Glucose lowering drugs | 4620 | 38.5 | 991 | 64.7 | 473 | 71.0 |
| Excluding insulins | 4538 | 37.8 | 978 | 63.8 | 458 | 68.8 |
| Biguanides | 4370 | 36.4 | 948 | 61.9 | 425 | 63.8 |
| Sodium-glucose co-transporter-2 inhibitors | 554 | 4.6 | 169 | 11.0 | 48 | 7.2 |
| Glucagon-like peptide-1 receptor agonists | 146 | 1.2 | 46 | 3.0 | 37 | 5.6 |
| Dipeptidyl peptidase-4 inhibitors | 2087 | 17.4 | 617 | 40.3 | 322 | 48.3 |
| Glitazones | 355 | 3.0 | 109 | 7.1 | 61 | 9.2 |
| Sulfonylureas | 1714 | 14.3 | 510 | 33.3 | 264 | 39.6 |
| Metiglinides | 41 | 0.3 | 15 | 1.0 | 6 | 0.9 |
| Glucosidase inhibitors | 256 | 2.1 | 98 | 6.4 | 54 | 8.1 |
| Insulins | 570 | 4.8 | 287 | 18.7 | 199 | 29.9 |
| Long acting | 431 | 3.6 | 221 | 14.4 | 158 | 23.7 |
| Intermediate acting | 220 | 1.8 | 116 | 7.6 | 82 | 12.3 |
| Fast acting | 186 | 1.6 | 72 | 4.7 | 58 | 8.7 |
| Premixed | 132 | 1.1 | 64 | 4.2 | 46 | 6.9 |
| **Bone medications** | **n** | **%** | **n** | **%** | **n** | **%** |
| [Bone Disease Medications](https://docs.wp3a.careme.mtg.pt/definitions/medications.html#bone-disease-medication) | 406 | 3.4 | 48 | 3.1 | 23 | 3.5 |
| [Vitamin D](https://docs.wp3a.careme.mtg.pt/definitions/medications.html#vitamin-d-and-analogues) | 721 | 6.0 | 171 | 11.2 | 176 | 26.4 |
| [Calcium](https://docs.wp3a.careme.mtg.pt/definitions/medications.html#calcium) | 616 | 5.1 | 94 | 6.1 | 50 | 7.5 |
| [Magnesium](https://docs.wp3a.careme.mtg.pt/definitions/medications.html#magnesium) | 1314 | 11.0 | 204 | 13.3 | 106 | 15.9 |
| [Estrogens and progestogens](https://docs.wp3a.careme.mtg.pt/definitions/medications.html#estrogens-and-progestogens) | 18 | 0.2 | 3 | 0.2 | 1 | 0.2 |
| [Calcitonins](https://docs.wp3a.careme.mtg.pt/definitions/medications.html#calcitonins) | 22 | 0.2 | 8 | 0.5 | 1 | 0.2 |

|  |  |  |  | **Persistent albuminuria categories, description, and range** | | | **Total per row** | **Overall CKD prevalence** |
| --- | --- | --- | --- | --- | --- | --- | --- | --- |
|  |  |  |  | **A1** | **A2** | **A3** |  |  |
|  |  |  |  | Normal to mildly increased | Moderately increased | Severely increased |  |  |
|  |  |  |  | < 30 mg/g  <3 mg/mmol | 30-300 mg/g  3-30 mg/mmol | ≥ 300 mg/g  ≥ 30 mg/mmol |  |  |
| **GFR categories (mL/min/1.73 m^2^), description and range** | **G1** | Normal or high | ≥ 90 | **Not applicable** | 0.2 | 0.0 | 0.2 | Not applicable |
|  | **G2** | Mildly decreased | 60-89 | **Not applicable** | 0.6 | 0.1 | 0.7 | Not applicable |
|  | **G3a** | Mildly to moderately decreased | 45-59 | 1.2 | 0.2 | 0.1 | 1.5 | There are 2.4 ≥ G3a but albuminuria was not available |
|  | **G3b** | Moderately to severely decreased | 30-44 | 0.6 | 0.2 | 0.1 | 0.9 |  |
|  | **G4** | Severely decreased | 15-29 | 0.2 | 0.1 | 0.1 | 0.4 |  |
|  | **G5** | Kidney failure | < 15 | 0.0 | 0.0 | 0.1 | 0.1 |  |
| **Total per column** | | | | 2.0 | 1.3 | 0.5 | **3.8%** | **6.2%** |

**Figure S1** – Risk of CKD progression/prognosis (%) by eGFR and albuminuria categories for females. Overall CKD prevalence is presented for all patients with two eGFR values < 60 ml/min/1.73 m^2^ (G3-G5) and/or two UACR values ≥30 mg/g (A2-A3) persistent for at least 3 months. From these, 3.8% of patients were possible to be stratified according to KDIGO guidelines and the CKD risk was defined as follow: green, low risk/no CKD in absence of markers of kidney disease; yellow, moderately increased risk; orange, high risk; red, very high risk. CKD, chronic kidney disease; eGFR, estimated glomerular filtration rate. According to KDIGO, patients in stage G1/A1 and G2/A1 were not characterized for CKD since other data of renal lesion, such as echography, urinary sediment and renal biopsy reports were not available. Data is presented for percentages over total female population.

|  |  |  |  | **Persistent albuminuria categories, description, and range** | | | **Total per row** | **Overall CKD prevalence** |
| --- | --- | --- | --- | --- | --- | --- | --- | --- |
|  |  |  |  | **A1** | **A2** | **A3** |  |  |
|  |  |  |  | Normal to mildly increased | Moderately increased | Severely increased |  |  |
|  |  |  |  | < 30 mg/g  <3 mg/mmol | 30-300 mg/g  3-30 mg/mmol | ≥ 300 mg/g  ≥ 30 mg/mmol |  |  |
| **GFR categories (mL/min/1.73 m^2^), description and range** | **G1** | Normal or high | ≥ 90 | **Not applicable** | 0.5 | 0.1 | 0.6 | Not applicable |
|  | **G2** | Mildly decreased | 60-89 | **Not applicable** | 0.8 | 0.2 | 1.0 | Not applicable |
|  | **G3a** | Mildly to moderately decreased | 45-59 | 0.5 | 0.3 | 0.1 | 0.9 | There are 0.8% ≥ G3a but albuminuria was not available |
|  | **G3b** | Moderately to severely decreased | 30-44 | 0.8 | 0.2 | 0.1 | 1.1 |  |
|  | **G4** | Severely decreased | 15-29 | 0.3 | 0.1 | 0.2 | 0.6 |  |
|  | **G5** | Kidney failure | < 15 | 0.2 | 0.0 | 0.1 | 0.3 |  |
| **Total per column** | | | | 1.8 | 1.9 | 0.8 | **4.5%** | **5.3%** |

**Figure S2** – Risk of CKD progression/prognosis (%) by eGFR and albuminuria categories for males. Overall CKD prevalence is presented for all patients with two eGFR values < 60 ml/min/1.73 m^2^ (G3-G5) and/or two UACR values ≥30 mg/g (A2-A3) persistent for at least 3 months. From these, 4.5% of patients were possible to be stratified according to KDIGO guidelines and the CKD risk was defined as follow: green, low risk/no CKD in absence of markers of kidney disease; yellow, moderately increased risk; orange, high risk; red, very high risk. CKD, chronic kidney disease; eGFR, estimated glomerular filtration rate. According to KDIGO, patients in stage G1/A1 and G2/A1 were not characterized for CKD since other data of renal lesion, such as echography, urinary sediment and renal biopsy reports were not available. Data is presented for percentages over total male population.
